# A Carbon Capture and Utilization Process for the Production of Solid Carbon Materials from Atmospheric CO_2_ – Part 2: Carbon Characterization

**DOI:** 10.1002/cssc.202401780

**Published:** 2024-11-19

**Authors:** Neele Uhlenbruck, Benjamin Dietrich, Stefan Heißler, Christoph M. Hofberger, Ralf Krumholz, Leonid Stoppel, Vanessa Trouillet, Peter G. Weidler, Thomas Wetzel

**Affiliations:** ^1^ Institute for Thermal Energy Technology and Safety Karlsruhe Institute of Technology Hermann-von-Helmholtz-Platz 1 76344 Eggenstein-Leopoldshafen Germany; ^2^ Institute of Thermal Process Engineering Karlsruhe Institute of Technology Kaiserstr. 12 76131 Karlsruhe Germany; ^3^ Institute of Functional Inferfaces Karlsruhe Institute of Technology Hermann-von-Helmholtz-Platz 1 76344 Eggenstein-Leopoldshafen Germany; ^4^ Institute for Applied Materials (IAM-ESS) and Karlsruhe Nano Micro Facility (KNMFi) Karlsruhe Institute of Technology Hermann-von-Helmholtz-Platz 1 76344 Eggenstein-Leopoldshafen Germany

**Keywords:** Carbon, Carbon storage, CO_2_ utilization, Methane pyrolysis, Liquid metal

## Abstract

This article is the second part of a study reporting the results of a novel carbon capture and utilization (CCU) process, which converts atmospheric CO_2_ into solid carbon materials. The CCU process combines direct air capture (DAC) with catalytic methanation, which is then followed by methane pyrolysis in a reactor filled with liquid tin. While Part 1 discussed the performance of the overall process and individual process steps regarding conversions and yields, Part 2 characterizes the solid carbon products obtained under various synthesis conditions. The effects of the pyrolysis temperature and the composition of the gas mixture from the methanation step on the solid carbon product are analyzed. Carbon powder is synthesized from methane with either N_2_, H_2_ or CO_2_ as most important impurity. Carbon samples are characterized using SEM, TEM, Raman spectroscopy, XPS and XRD analysis. Very thin, disordered carbon flakes, soot aggregates and large carbon onions are identified as the main solid products of the process. Their formation mechanisms in the liquid metal‐filled pyrolysis reactor are discussed.

## Graphical abstract

Carbon samples synthesized via methane pyrolysis in a bubble column reactor filled with liquid tin are characterized. Formation mechanisms are discussed for the most prevalent carbon species (thin carbon flakes, carbon (nano−)onions and soot aggregates). The influence of the feed gas composition and pyrolysis temperature on the solid carbon product are analyzed.

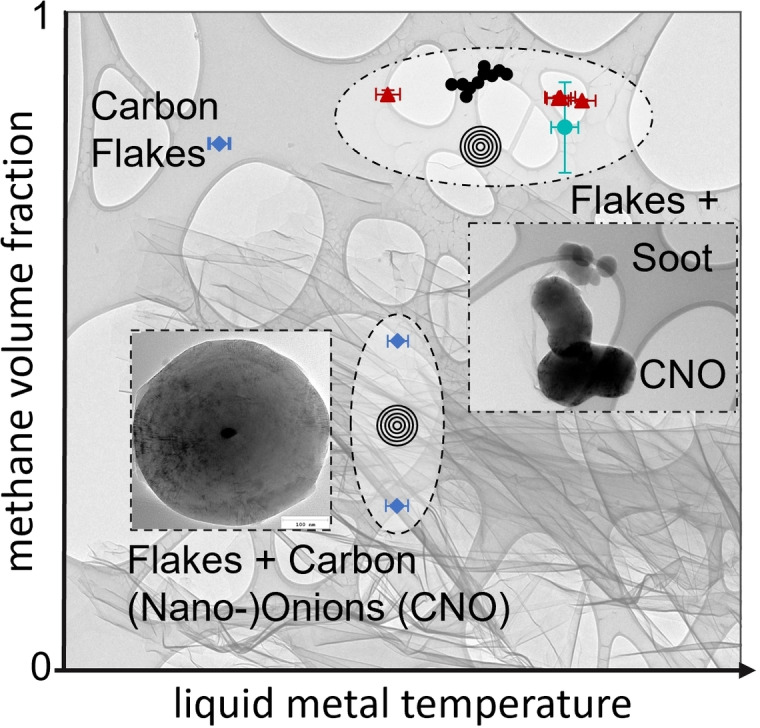

## 1 Introduction

Several recent studies[^1^, ^2^, ^3^] have analyzed and summarized the options of using CO_2_ as a resource for the production of chemicals and fuels, with the required energy provided by renewables. Regarding solid carbon products, significantly less research has been done. Molten carbonate electrolysis seems to be a promising approach, which has led to the synthesis of carbon nano‐onions (CNO),^[4]^ carbon nanotubes (CNT)[^5^, ^6^, ^7^] and other types of carbon.[^5^, ^8^, ^9^] Different synthesis routes use reducing metals (Mg,[^10^, ^11^, ^12^, ^13^, ^14^] Li,[^15^, ^16^] Ga,^[17]^ Ca^[11]^) or metal compounds (LiAlH_4_ ^[18]^) to convert CO_2_ into various solid carbon materials. These batchwise synthesis approaches end up with metal oxides or carbides, though, which would need regeneration, and face the challenge of continuous operation. On the other hand, many solid carbon materials are currently still produced from fossil precursors (e. g., carbon black,^[19]^ carbon fibres,^[20]^ hard carbon,^[21]^ synthetic graphite,^[22]^ which is also a precursor for graphene production^[23]^). To achieve a fossil‐free circular economy, research activities focusing on the production of solid carbon materials from CO_2_ should be extended to complement studies on biomass‐based carbon production, which focuses mainly on biochar.[^24^, ^25^, ^26^]

This article is the second of two articles reporting the successful synthesis of solid carbon powder from atmospheric CO_2_ via a novel continuous CCU process. Part 1^[27]^ focuses on the influence of process parameters and gas impurities on the overall process performance, providing details on conversions, product yields and by‐product formation. In short, the process catalytically converts atmospheric CO_2_ obtained via direct air capture (DAC) and hydrogen from a closed H_2_ loop into methane and water. Solid carbon powder is then obtained by thermally splitting methane into its elemental components, according to the simplified Equation 1.

(1) $$\begin{array}{c} CH_{4}\left(g\right)↔\;{2\;H}_{2}\;\left(g\right)+C\;\left(s\right)\\ \Delta H_{R}^{0}=74.8\;\;kJ/mol\;CH_{4} \end{array}$$

Hydrogen is recycled to the methanation step after methane pyrolysis and from water electrolysis, as elaborated in more detail in the first part of this study.^[27]^ A bubble column reactor filled with liquid tin is employed to prevent carbon deposition on the reactor walls.^[28]^ The liquid metal (LM)‐based pyrolysis reactor was already developed several years ago[^28^, ^29^] and recently, a successful scale‐up and the pyrolysis of natural gas have been reported.[^30^, ^31^] However, a comprehensive analysis of the produced carbon powder is missing to date with the previous research activities focusing on the decarbonization of methane/ natural gas for hydrogen production. Geißler et al.^[28]^ described the solid carbon as agglomerates of presumably spherical particles (40–100 nm) in the shape of flakes based on scanning electron microscopy (SEM) images. Their XRD analysis revealed a low crystallinity.

Zhang et al.^[32]^ give a comprehensive overview of studies on methane pyrolysis in various molten media. Their review shows that a significant influence of the employed molten phase on the carbon formation mechanism and final product has to be assumed. This is in agreement with observations made by Ding et al.^[33]^ who synthesized graphene via chemical vapor deposition on various molten metal substrates and noticed a strong influence of the substrate on the graphene quality. Besides, if the molten substrate is catalytically active, catalysis might change the predominant carbon formation mechanism. Carbon solubility in the molten medium is another factor to consider. Therefore, the carbon characterization and conclusions presented in this article might not be transferable to other molten media‐based pyrolysis reactors. Vice versa, the results regarding the carbon products synthesized in other molten media^[32]^ cannot be transferred to the carbon obtained in molten tin without further examination.

A more recent preliminary transmission electron microscopy (TEM) analysis^[34]^ of the carbon produced by Geißler et al.^[28]^ revealed not only thin flakes (not flake‐lake agglomerates of spherical particles, as proposed earlier^[28]^) but also soot aggregates distributed among very thin flakes, which resembled graphene. Conclusions regarding the (dis)order and defect density of those flakes could not be drawn based on the preliminary analysis, though. This article answers the question, which types of carbon are formed during methane pyrolysis in a bubble column filled with liquid metal by giving a detailed characterization of various carbon samples. Furthermore, it analyzes which mechanisms are relevant for the synthesis of the prevailing types of carbon when tin is used as molten medium. The third question addressed is how the synthesis conditions, especially temperature and initial methane concentration, influence the formation of solid carbon. Previously, the correlation between pyrolysis conditions and synthesized types of carbon could not be analyzed as the carbon accumulated and mixed on top of the LM surface over the course of several hydrogen production experiments.[^28^, ^30^, ^31^] The latter obstacle was successfully overcome in this study by introducing a carrier gas above the LM surface for the continuous removal and subsequent downstream collection of the carbon powder. The synthesis conditions of the various carbon samples discussed in this article are therefore known.

All in all, the carbon characterization reported in this article is not only valuable in the context of a circular economy and CO_2_ utilization to replace fossil‐based carbon products. Also, with regards to natural gas decarbonization as a transition technology to produce turquoise hydrogen, a better understanding of the carbon properties and mechanisms leading to carbon formation is important to tune the carbon (by−)product quality. In the latter case, special attention has to be paid to the storage potential of possible applications, though, to ensure the utilization of the carbon product does not result in delayed fossil emissions. This is taken into consideration when potential applications for the carbon powder are discussed in the final section of this article.

## 2 Experimental

### 2.1 Carbon Synthesis

**Figure 1:**
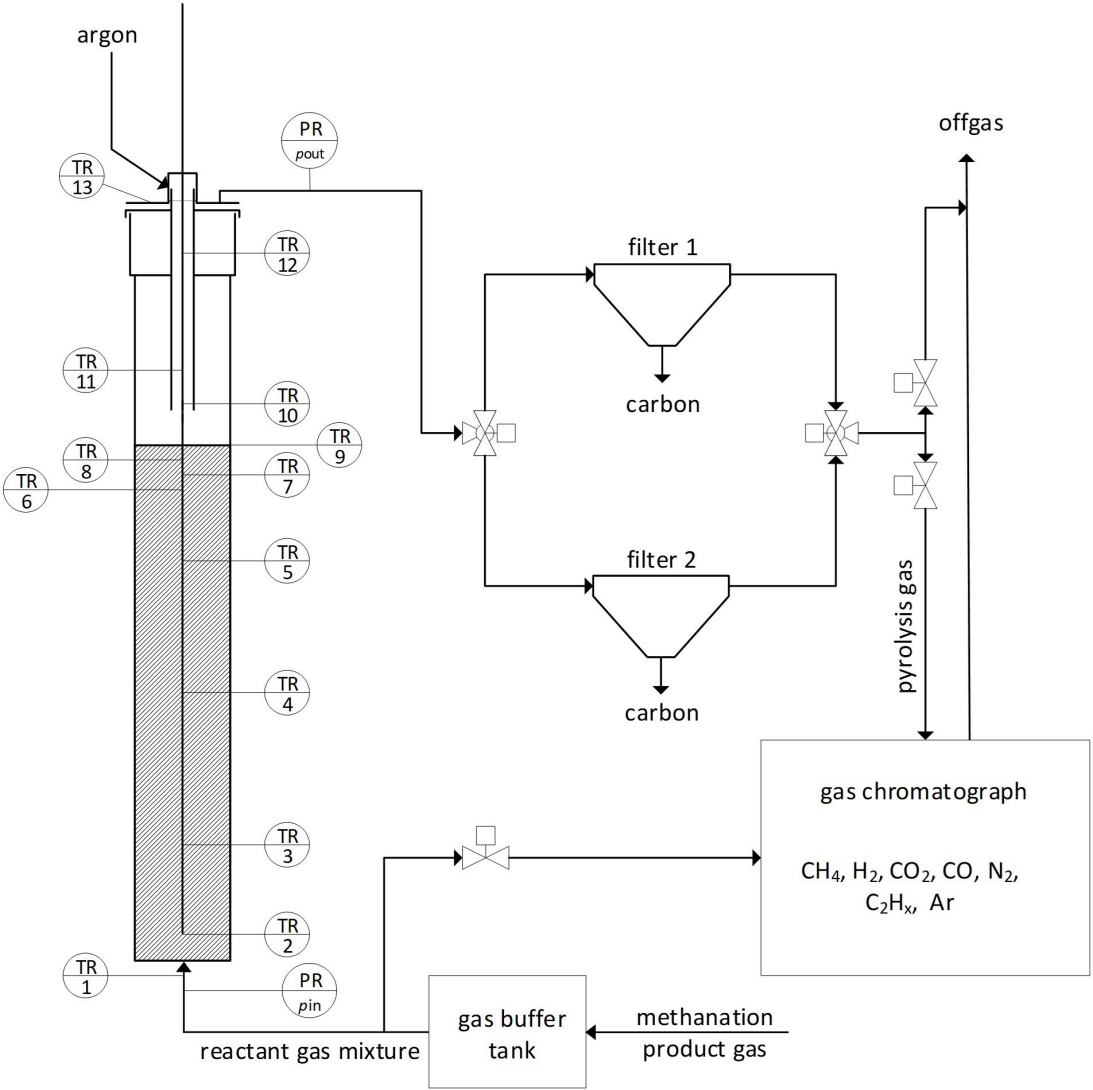
Simplified process flow diagram of the pyrolysis facility (as given in Figure S2 in the supplementary information (SI) of Part 1^[27]^)

Solid carbon powder was obtained by thermally splitting methane in a bubble column reactor filled with liquid tin. The pyrolysis reactor (a quartz tube with an inner diameter of *d*_i*=*_40 mm and a length of *L*=1300 mm) is heated by an electric tube furnace and filled with liquid tin (99.99 %) up to a height of approximately 1020 mm. The feed gas enters the reactor through a single orifice (*d*_O_=0.5 mm) at the bottom. An additional carrier gas stream (Ar 4.8) is introduced in the top section of the reactor via a tube (stainless steel 1.4571 for experiments #7 and #8, Al_2_O_3_ for all other experiments) ending just above the LM surface to continuously remove the carbon powder. The effects of argon addition and details about the carbon removal are given in part 1.^[27]^ The fine powder is then separated from the gas in filters further downstream. The gas mixture leaving the reactor is analyzed by gas chromatography. A simplified process flow diagram of the pyrolysis facility is depicted in Figure 1.

Several thermocouples (TC) along the axis of the reactor record the temperature of the LM *T*_LM_ (bottom section, TR2 to TR9) and of the gas (upper section, TR10 to TR12). The average LM temperature ${\bar{T}}_{LM}$ and further pyrolysis process parameters (pressures, gas flow rates) are given in Table 1. ${\bar{T}}_{LM}$ represents the average of TR2 to TR9 over the period of the respective experimental run. A representative temperature profile and details on measurement uncertainties are given in the supplementary information (SI) of part 1.^[27]^

**Table 1:** Pyrolysis parameters (as given in Table S3 of the SI of Part 1^[27]^).

Experiment	${\bar{T}}_{LM}$ /°C	${\bar{p}}_{in}$ /bar(a)	${\bar{p}}_{out}$ /bar(a)	${\dot{V}}_{reac}$ /ml_N_ min^−1^	${\dot{V}}_{Ar}$ /l_N_ min^−1^	Feed composition
#1	1098.0±11.9	1.779±0.062	1.015±0.030	364.0±25.6	9.000±0.047	See Table 2
#2	1096.6±14.0	1.831±0.186	1.083±0.021	354.9±6.9	9.000±0.047	See Table 2
#3	1096.2±9.6	1.840±0.050	1.089±0.011	358.0±10.1	9.000±0.047	See Table 2
#4	1045.6±10.1	1.815±0.011	1.085±0.009	353.5±5.4	9.000±0.047	See Table 2
#5	1048.2±9.5	1.752 ±0.004	1.075 ±0.008	350.0±1.8	9.000±0.046	CH4:N2–25 : 75
#6	1048.5±9.4	1.770 ±0.052	1.075 ±0.008	350.0±1.9	9.000±0.046	CH4:N2–50 : 50
#7	995.6±15.8	1.729±0.005	1.084±0.008	200.0±1.0	12.0±0.06	CH_4_:N_2–_80 : 20
#8	995.7±8.6	1.728±0.004	1.083±0.008	12.0±1.0	12.0±0.06	CH_4_:N_2f_–80 : 20

Part 1^[27]^ describes the arrangement of the TCs and the setup of the pyrolysis test facility in more detail. Information on the experimental setup of the coupled CCU process and the methanation step can also be found in Part 1.^[27]^ The following Table 2 gives the compositions of the gas mixtures fed into the pyrolysis reactor for carbon synthesis during the coupled operation of the CCU process (experiments #1 to #4). The molar fraction ${\tilde{y}}_{div}$ comprises gases that were not individually quantified. The major component is probably N_2_, but also Ar, O_2_ and/or H_2_O could be part of ${\tilde{y}}_{div}$ as elaborated in Part 1.^[27]^ During experiments #1 to #4, the gas mixtures were synthesized from atmospheric CO_2_ in the methanation step preceding the pyrolysis step. Four further pyrolysis experiments, #5 to #8, were conducted independently with the respective ratios of CH_4_:N_2_ given in Table 1. The gases (CH_4_ 4.5 and N_2_ 5.0) for these additional experiments were provided from gas bottles. Details on the gas analyses and the calculation of the uncertainties of the feed gas compositions for experiments #1 to #4 given in Table 2 are provided in Part 1^[27]^ and the respective SI.

**Table 2:** Average pyrolysis feed gas composition obtained from methanation of atmospheric CO_2_ during coupled process operation (as given in Table S4 of the SI of Part 1).^[27]^ Experimental runs #1 to #4 correspond to #1 to #4 in Table 1.

Experiment	${\tilde{y}}_{CH4}$ /%	${\tilde{y}}_{H2}$ /%	${\tilde{y}}_{CO2}$ /%	${\tilde{y}}_{CO}$ /%	${\tilde{y}}_{div}$ /%
#1	82.46± 6.85	10.07± 5.77	0.06± 0.16	0.01± 0.08	7.40± 12.86
#2	87.01± 0.29	1.18± 0.22	4.31± 0.59	0.01± 0.08	7.49± 1.21
#3	86.85± 0.34	1.18± 0.29	4.03± 0.81	0.01± 0.08	7.93± 1.55
#4	87.4± 0.62	0.97± 0.35	5.56± 1.56	0.02± 0.09	5.96± 2.62

### 2.2 Carbon Characterization

*(High Resolution) Transmission Electron Microscopy ((HR)TEM)* was done with a Philips CM200 FEG microscope with an acceleration voltage of 200 kV and *Scanning Electron Microscopy (SEM)* was done with a LEO 1530 Gemini microscope at an acceleration voltage of 5 kV. For (HR)TEM and SEM examination, a random sample of the carbon produced in each experiment was taken and dispersed in isopropanol, then transferred onto copper grids with lacey carbon films. SEM and (HR)TEM images were analyzed with the software ImageJ 1.52 v to determine fringe lengths and the particle size distributions (PSD) of primary soot particles. For the PSDs of the samples, the diameters of primary soot particles were measured using ImageJ.

*Raman Spectroscopy* Raman spectra were obtained with a Bruker Senterra 2 Raman spectrometer with a laser ($\lambda_{L}\;$ =532 nm, operated at 6.25 mW, diameter of irradiated area 5 μm) as excitation source. The integration time for each spectrum was 20 s with four coadditions (4x5 s). The first order Raman spectra were fitted with five peaks according to the methodology described by Sadezky et al.^[35]^ for disordered carbon but with peak positions limited to the respective ranges they indicated for soot. The second order Raman bands were fitted independently with three Lorentzian peaks. The Raman spectra of samples C#7 and C#8, depicted in Figure 4 and Figure S8 in the SI, display averaged values of Raman spectra measured at 28 (C#7) and 26 (C#8) different sample positions.

*X‐ray diffraction (XRD)* analysis was done with a Bruker D8 diffractometer with Cu radiation (CuK_α1,2_ 0.154060 nm) equipped with a position sensitive detector Lynxeye running from 5° to 95° 2θ with 2 s per step resulting in a total counting time per step of 384 s. Data was evaluated with the Bruker software DIFFRAC.EVA V5.2 and TOPAS V6.^[36]^

*X‐ray Photoelectron Spectroscopy (XPS)* measurements were performed using a K‐Alpha+ XPS spectrometer (ThermoFisher Scientific). The Thermo Avantage software was used for data acquisition and processing. All samples were analysed using a microfocused, monochromated Al Kα X‐ray source (400 μm spot size). The K‐Alpha+ charge compensation system was employed during analysis, using electrons of 8 eV energy, and low‐energy argon ions to prevent any localized charge build‐up. The spectra were fitted with one or more Voigt profiles (BE uncertainty: ±0.2 eV) and Scofield sensitivity factors^[37]^ were applied for quantification. All spectra were referenced to the graphitic sp^2^ C 1s peak at 284.4 eV binding energy controlled by means of the well‐known photoelectron peaks of metallic Cu, Ag, and Au, respectively.

## 3 Results and Discussion

The carbon powder of most samples was found to consist of several types of solid carbon, which are described in the following Section 3.1. After the characterization of the various types of carbon, the influence of the synthesis conditions on the composition of the carbon product will be discussed in Section 3.2. Based on Section 3.2 and literature, hypotheses regarding the formation mechanisms of the prevailing types of carbon are presented in Section 3.3. Section 3.4 discusses possible applications for the solid carbon product.

### 3.1 Carbon Characterization

**Figure 2:**
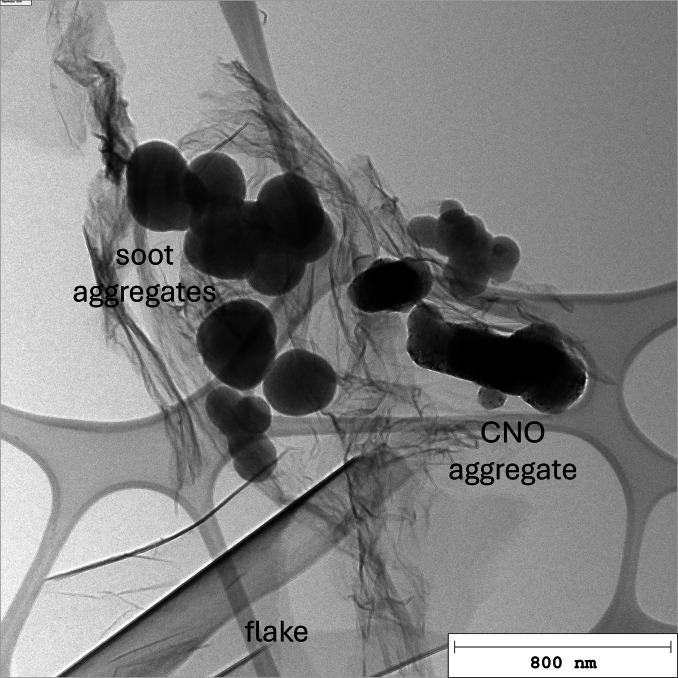
TEM image showing the three most prevalent types of carbon: thin flakes, soot aggregates and carbon (nano−)onions (CNO).

Analysis of the carbon samples via Transmission Electron Microscopy (TEM) revealed various types of carbon, which will be described in detail in the following subsections. The main types of carbon, which are depicted in Figure 2, were found to be thin carbon flakes, soot aggregates and carbon (nano−)onions (CNO).

Table 3 gives an overview of the types of carbon found in the samples. The sample numbers C#1 to C#8 correspond to the experimental synthesis conditions given in Table 1 with matching numbers (#1 to #8). As shown in Table 3, thin carbon flakes were present in all samples analyzed. Their properties are described in Section 3.1.1. Soot aggregates, which were found in some samples only, are characterized in Section 3.1.2. CNO, which are approximately spherical particles consisting of a concentrical layered structure, are presented in Section 3.1.3. While flakes, CNO and soot aggregates appear to be the most prevalent types of carbon, further carbon morphologies were found in some samples. They are described in Section 3.1.4. Section 3.1.5 addresses tin nanoparticles that were found to cover the surface of some carbon flakes and soot aggregates.

**Table 3:** Types of carbon identified by TEM and/or SEM analysis in samples synthesised under different process conditions. The sample numbers C#1 to C#8 correspond to the experimental synthesis conditions given in Table 1 with matching numbers (#1 to #8). ✓ indicates a type of carbon was identified, while–indicates that no specimen of the respective type of carbon was found. ? marks uncertain observations.

Sample	Smooth flakes	Islands on flakes	Large CNO	Small CNO clusters	Soot aggregates	Microbes	Nano‐Sn
C#1	✓	✓	✓	✓	✓	‐	✓
C#2	✓	✓	✓	‐	✓	✓	✓
C#3	✓	✓	✓	‐	✓	✓	✓
C#4	✓	✓	✓	✓	✓	✓	✓
C#5	✓	✓	✓	–	–	✓	✓
C#6	✓	✓	✓	?	–	✓	✓
C#7	✓	?	–	?	–	–	✓
C#8	✓	?	–	?	–	–	✓

#### 3.1.1 Carbon Flakes

**Figure 3:**
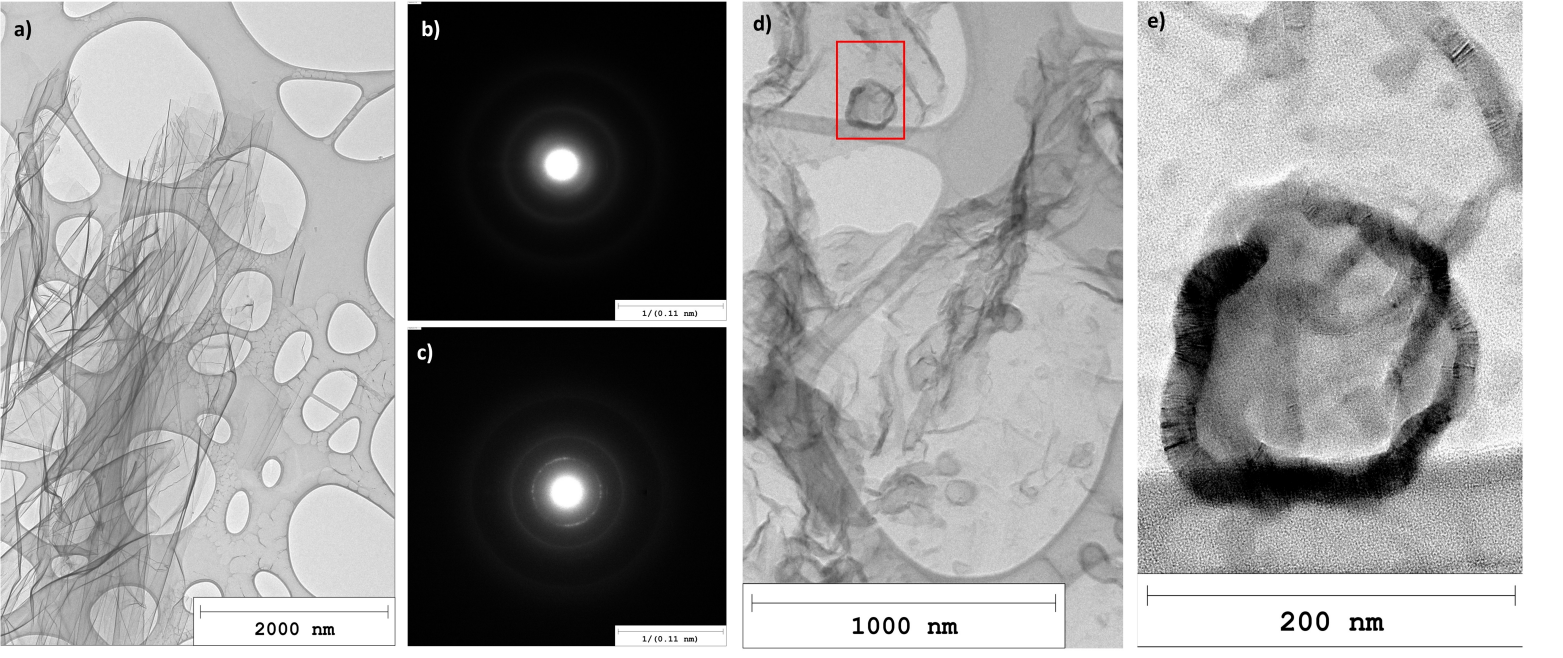
TEM images of thin carbon flakes found in the pyrolytic carbon samples: (a) very thin pyrocarbon flakes with smooth surface and a typical SAED pattern of those flakes (b). (d) Carbon flakes with “islands”, roughly circular graphitic structures and a typical SAED pattern of those flakes (c). (e) close‐up of the “island” marked by the red box in (d).

Figure 3a shows very thin graphene‐like carbon flakes with a typical selected area electron diffraction (SAED) pattern depicted in Figure 3b. Those smooth flakes are ubiquitous, and many have lateral dimensions of several microns.

However, the very diffuse SAED image of such a smooth flake, shown in Figure 3b, suggests a rather amorphous nature of the carbon, in contrast to the crystalline nature of graphene and graphite. High resolution transmission electron microscopy (HRTEM) images, such as Figure S1 in the SI, show the turbostratic arrangement of carbon layers with mostly short fringe lengths of about 1.3±0.6 nm.

While the surface of most carbon flakes appears to be creased but rather homogeneous, some flakes, e. g., the one depicted in Figure 3d, are sprinkled with roughly circular structures. SAED analysis of those carbon flakes (Figure 3c) reveals a more crystalline nature than for the plain flakes, indicated by the better defined graphitic (002) diffraction circle. Figure 3e shows the magnified HRTEM image of the structure marked by a red box in Figure 3d. The Moiré patterns along the edges of the structure indicate ordered layers of carbon with slightly shifted angles. The graphitic layering in those regions is also observed on further magnified HRTEM images. From a 3D perspective, these approximately circular structures (in the 2D TEM plane) should probably rather be regarded hemispheres and presumably correspond to the dotted surfaces observed for some carbon flakes during Scanning Electron Microscope (SEM) analysis. An example of such a rough surface with approximately hemispherical structures is shown in Figure S4 in the SI, where the background also shows another flake with a rather smooth surface, which is typical of most carbon flakes. The diameters of the circular structures seen with TEM and the hemispherical structures observed with SEM lie in a similar range, as listed in Table S1 in the SI. For this comparison only the larger structures indicated in Figures S3 and S4 were analyzed. In both TEM and SEM images, there are also many smaller features visible, though, such as the small, dark spots seen in Figure 3e. For filled carbon hemispheres or spheres, a decrease of brightness towards the thicker center would be expected in TEM images. This is observed for example in Figure 2, where the outer edges of the depicted carbon onions and primary soot particles are brighter than their centers. An analysis of the grey value across some of the structures from Figure 3d, which is shown in Figure S3, reveals slightly darker but relatively constant shades of grey for the centers of the circular structures compared to the surrounding carbon flake. This indicates a thicker carbon layer and thus reduced transmission of the electron beam compared to the carbon flake. The edges of the structures showing curved graphitic carbon layers are darker, though, than the centers. The grey level between the dark edges does not appear to increase towards the center. Therefore, if the roughly circular structures found on some TEM images correspond to the approximately hemispherical structures seen on some SEM images, the latter might be hollow hemispheres with graphitic shells sitting on top of the carbon flakes.

**Figure 4:**
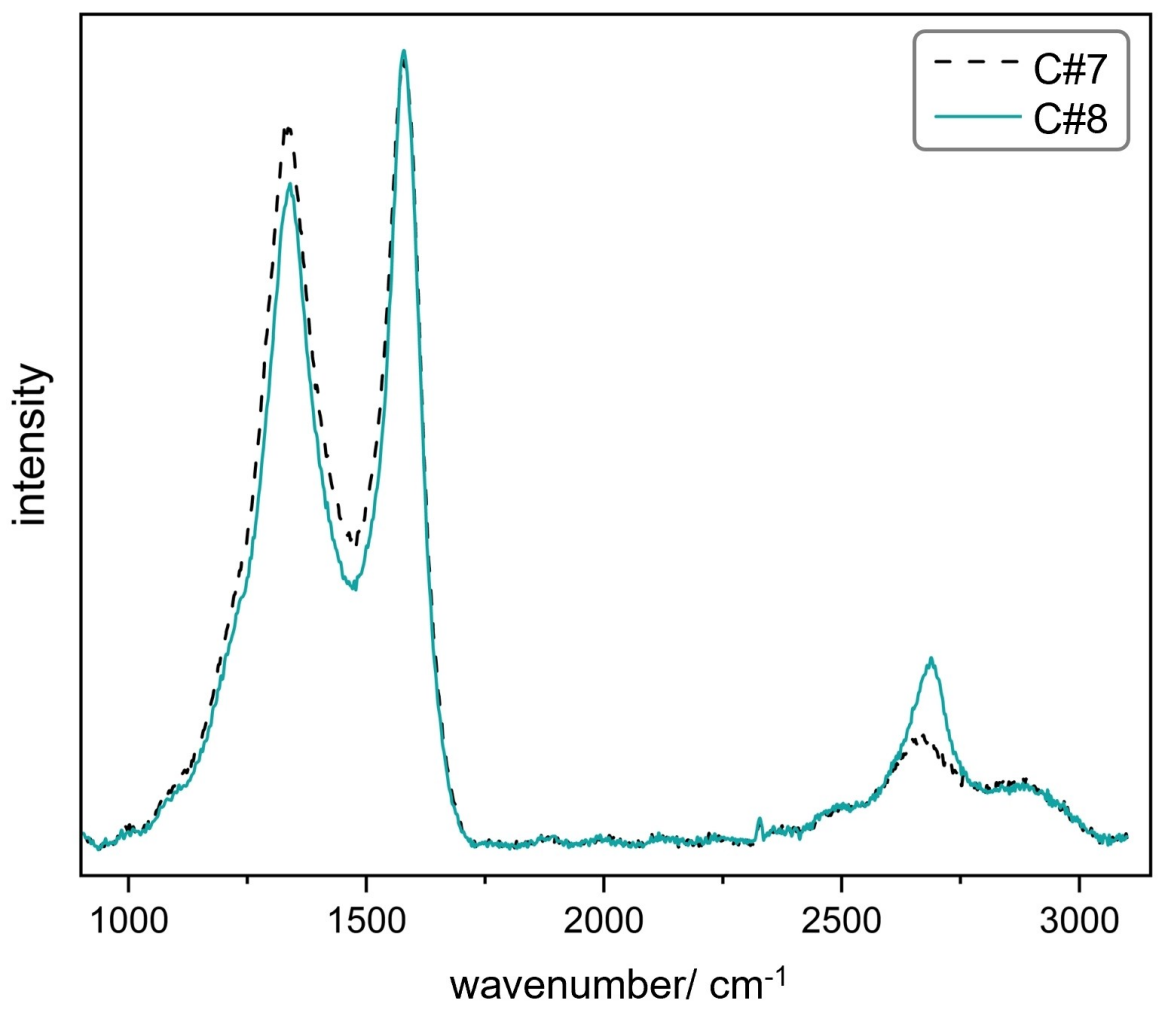
Averaged Raman spectra of samples C#7 and C#8.

The disordered nature of the flakes can also be seen in their Raman spectra shown in Figure 4. To obtain meaningful information about the carbon flakes form their Raman spectra, samples C#7 and C#8, both synthesized at 1000 °C, were analyzed as these are the only samples where no other types of carbon were identified by TEM analysis. A comparison of the XRD diffractograms of C#8 and C#3 (Figure S7 in the SI) supports this further. While there are only very weak carbon signals for C#8, which are ascribed to the disordered pyrocarbon flakes observed during TEM analysis, the diffractogram of C#3 shows a more pronounced profile of disordered carbon. The stronger carbon signals likely originate from the other carbon species (CNO, soot), which are present in C#3, thus adding to the weak XRD signal of the flakes. The diffractogram of C#8 exhibits hardly any carbon signals, which is a further indicator for the lack of other carbon species in the sample. Hence, the averaged first order Raman spectra of C#7 and C#8 depicted in Figure 4 and the corresponding characteristic figures given in Table 4 can be attributed to the flakes only and do not have to be interpreted as an overlay of Raman signals originating from various types of carbon. In contrast, all the other samples listed in Table 3 comprise various types of carbon, the ratios of which are currently not quantified and probably dependent on synthesis conditions. The Raman spectra of those other samples are therefore expected to be overlays of Raman spectra corresponding to the individual types of carbon, thus rendering their interpretation difficult. For example, a perceived increase of defects (shift of the intensity ratio of D and G band *I*_D_/*I*_G,_ larger full width at half maximum of the D band (FWHM_D_)) in one of those spectra could be attributed to more defects in the thin carbon flakes but also to a larger amount of disordered soot particles. An unambiguous interpretation of those spectra is therefore currently not possible, and the following discussion is limited to the carbon flakes found in C#7 and C#8.

**Table 4:** Characteristics of averaged Raman spectra of samples C#7 and C#8.

Sample	Pos. D/cm^−1^	FWHM_D_/cm^−1^	Pos. G/cm^−1^	FWHM_G_/cm^−1^	Pos. 2D/cm^−1^	FWHM_2D_/cm^−1^	*I*_D_/*I*_G_	*I*_2D_/*I*_G_
C#7	1335	136	1569	64	2667	170	1.44	0.21
C#8	1339	133	1574	57	2675	130	1.15	0.22

In agreement with the previously discussed HRTEM and SAED analyses of the thin carbon flakes, the Raman spectra reveal the high degree of disorder in the carbon lattice, marking the thin flakes as clearly distinct from crystalline graphene or graphite on the atomic scale. The relatively low position of the G band and the *I*_D_/*I*_G_ ratios of 1.15 and 1.44 of the two samples indicate a mixture of nanocrystalline graphite and amorphous carbon, located in stage 2 of Ferrari and Robertson's amorphization trajectory.^[38]^ They also proposed a correlation to estimate the crystallite size *L*_a_ in that stage from the *I*_D_/*I*_G_ ratio to complement the Tuinstra Koenig correlation,^[39]^ which is no longer applicable when *L*_a_ falls below 2 nm. Applying Ferrari and Robertson's criterium,^[38]^ *L*_a_ can be estimated to range from about 1.2 to 1.6 nm for the two samples, which is in good agreement with the fringe lengths reported above.

Furthermore, a good fit of the measured Raman spectra (see Figure S8a in the SI) requires a peak at 1500 cm^−1^, which is associated with amorphous carbon.[^35^, ^38^, ^40^] The broad D band, originating from breathing modes of aromatic rings, indicates a high defect density including heptagons and pentagons and a wide size distribution of aromatic clusters.^[38]^ An additional indicator of disorder is the shoulder of the D band. However, the presence of a distinguishable 2D band also shows a certain degree of order in the carbon lattice. As described by Sadezky et al.^[35]^ for some soot samples, the D+G band at about 2900 cm^−1^ is the second most prominent feature of the comparatively weak second order Raman modes of the carbon flakes. The averaged Raman spectrum of sample C#7, which was synthesized under the same pyrolysis conditions as C#8, has a slightly less pronounced 2D band and higher *I*_D_/*I*_G_ ratio, which indicates a slightly higher degree of disorder in stage 2 carbons.^[38]^ Otherwise, C#7 shows all the same features described for C#8. The observed differences might be due to the progression of steel corrosion that was noted after the experiments by visual inspection of the argon feed tube. Traces of other metals (steel species) in the LM show up in the XRD diffractogram of C#8 (Figure S7 in the SI) as small peaks next to intense tin peaks. These small peaks are missing in the diffractogram of C#3 (also given in Figure S7 in the SI), which was synthesized during a different set of experiments with a ceramic argon feed tube instead of a steel tube. The increasing amount of iron traces could have induced a slightly more ordered structure of C#8, which was synthesized after C#7, since iron is a catalyst used to grow (ordered) MWCNT^[41]^ or CNO.^[42]^ GC analysis of the pyrolysis gas during the synthesis of samples C#7 and C#8 did not indicate any differences caused by the onset of corrosion and no corrosion was noted during or after the synthesis of the other samples listed in Table 3. Considering the results of HRTEM, SAED and Raman analysis, the texture of the thin carbon flakes resembles pyrocarbon, which is usually deposited via CVD of hydrocarbons on solid surfaces, e. g., as a biocompatible coating for medical applications.^[43]^

#### 3.1.2 Soot Aggregates and CNO

**Figure 5:**
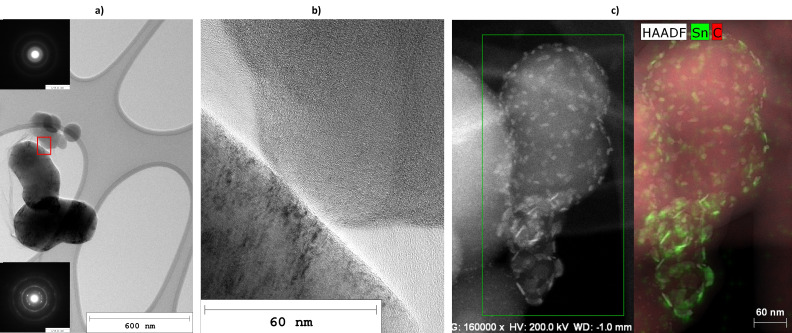
TEM images of several types of carbon found in the pyrolytic carbon samples: a soot aggregate (top) with the upper SEAD pattern corresponding to soot and an aggregate of large CNO (bottom) with the SEAD pattern at the bottom corresponding to CNO (a), close‐up of the region marked by a red box in a) showing the typical structure of CNO and soot (b), HAADF image of soot particles with tin nanoparticles scattered across the surface (c).

Figure 5a shows a soot aggregate (top) in proximity of an aggregate of carbon (nano−)onions (CNO, bottom). The inlayed representative SAED patterns of these distinct types of particle aggregates show the disordered structure of soot in contrast to the ordered one of CNO. The HRTEM image in Figure 5b, which is a magnification of the area in the red box of Figure 5a, also displays the different structures. While short and curved fringes show inside the soot particle, long coherent carbon layers arranged in concentrical spheres can be seen on the edge of the CNO. Typical soot aggregates were identified in all carbon samples (C#1 to C#4) synthesized from atmospheric CO_2_ during the coupled process operation.

Table 5 gives the particle size distribution (PSD) of the primary particles that form soot aggregates. Taking the standard deviations of the average particle diameter $\bar{d}$ into consideration, there is no significant difference between the soot particles produced under the various pyrolysis conditions. The standard deviation of the average diameter is high and the difference between *d*_10_ and *d*_90_ large, typical of a broad PSD. For all pyrolysis conditions investigated, the average diameter of the primary soot particles is larger than 100 nm. Compared with industrial carbon blacks, the PSD fits the range of large carbon black varieties.

**Table 5:** Primary soot particle size distributions (PSD). The number of primary particles that were analysed to obtain the PSD of each sample is given by n .

Sample	$\bar{d}$ /nm	$d_{10}$ / nm	$d_{50}$ /nm	$d_{90}$ /nm	n
C#1	142±69	40	138	234	119
C#2	133±30	96	131	170	94
C#3	169±72	78	161	258	498
C#4	159±23	138	157	190	15

The average primary particle sizes of the samples correspond best to the typical particle size of the formerly produced “fine thermal” carbon black (120 to 200 nm^[19]^ or 180 to 200 nm^[44]^). The latter was obtained via the pyrolysis of natural gas diluted with inert gas.^[19]^

While some CNO form aggregates like the ones shown in Figure 5a, also single particles are found, e. g., the ones displayed in Figure S6 in the SI. The CNO tend to have larger diameters than soot particles, often reaching several hundreds of nanometers. Compared to most CNO reported in other studies this is remarkably large and the classification as “nano” might even be debatable. In the context of (catalytic) hydrocarbon pyrolysis and CNO formation via CVD, reported CNO diameters range from 5 to 90 nm.[^45^, ^46^, ^47^, ^48^] Large CNO of several hundreds of nanometers in diameter are hardly mentioned. Two examples of other large CNO were obtained via different synthesis routes, i. e., by exposing amorphous soot samples to intense electron beam irradiation^[49]^ or by annealing diamonds of similarly large sizes.^[50]^

Due to the large diameters of the CNO in our samples, it is difficult to visualize their cores. Some, for example the one displayed in Figure S6b in the SI, contain a tin core, which shows as a dark spot in the center on the TEM image. Very few CNO that appear to have a hollow core were also found, indicated by a small bright spot with sharp outline in the center of those CNO. However, most CNO small enough to be accessible to TEM observation do not appear to have hollow or tin cores. One of them is shown in Figure S6a in the SI, where HRTEM analysis reveals a probably disordered, roughly ellipsoidal core with a diameter of about 15 to 25 nm and ordered carbon layers around it. For one of the few other “small” CNO detected during TEM analysis, the layers continue almost to the center, leaving only 3 to 4 nm as a core without clear layer structure. The prevailing core structure (amorphous or layered) remains an open question, however, as hardly any CNO small enough for a clear visualization of their center were found.

#### 3.1.3 Further Types of Carbon

**Figure 6:**
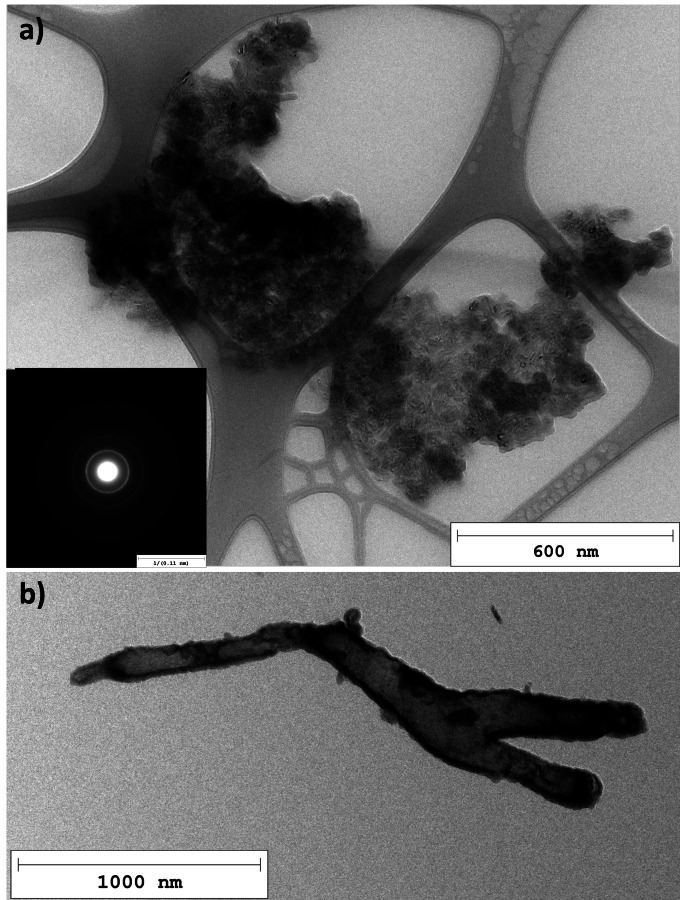
TEM images of a cluster of structures resembling small carbon nano‐onions (CNO) with a typical SAED pattern (a) and of a carbon “microbe” (b).

On top of the various types of carbon mentioned so far, the samples also contain clusters of small, ordered carbon structures shown in Figure 6a. The distinct outline of the SAED pattern as well as the Moiré effect and HRTEM analysis (Figure S5b in the SI) reveal the ordered arrangement of several carbon layers. These clusters appear to consist of spherical and elongated carbon structures resembling small CNO with only few layers and short multi‐walled carbon nanotubes (MWCNT), although distinct features are hard to distinguish.

Finally, a type of carbon we are going to call “carbon microbe” in the following discussion, which is shown in Figure 6b, was identified in several samples. The edges of those carbon microbes show relatively ordered layered carbon and cause the Moiré effect. The one shown in Figure 6b branches out on one end, but others having several branches or no branches at all were also found. They seem to be mainly closed structures (example shown on the SEM image Figure S2 in the SI), as opposed to rolled up graphitic carbon flakes.

#### 3.1.4 Tin Particles

In all samples, roughly spherical tin particles with diameters in the range of few to several hundreds of microns are found. They originate from the bursting of bubbles when they reach the tin surface. The tin particles were carried out of the reactor together with the carbon powder due to the high carrier gas streams applied during the synthesis experiments. If the inert carrier gas stream is not additionally intended to quench the pyrolysis reactions above the LM surface (see part 1^[27]^), its volume flow could probably be reduced in future experiments. If carbon recovery is the only objective, an improved carbon‐tin separation could likely be achieved by optimizing the carrier gas flow. As the carbon density is a lot lower than the density of tin, the removal of the carbon particles from the reactor should require much lower gas velocities than the entrainment of tin microparticles. In addition to tin microparticles, soot aggregates and carbon flakes were found that are dotted with tin nanoparticles, as shown in Figure 5c. The presence of nanoscale tin particles is further supported by Raman analysis. In addition to the typical Raman bands expected for pyrocarbon, some individual spectra also showed intermediate frequency phonon modes (IFM) up to 1000 cm^−1^ (Figure S8b in the SI). Carbon nanomaterials such as carbon nanotubes (CNT)^[51]^ or CNO^[52]^ are known to have Raman active phonon modes in that range. The typical wavenumbers reported for those materials do not match the spectrum shown in Figure S8b, though. It resembles most closely a spectrum of SnO_2_ nanoparticles recorded by Bonu et al.^[53]^ They concluded that nanoscale dimensions of the particles activate additional Raman modes that are otherwise forbidden in bulk materials. As tin nanoparticles, which we identified by HAADF on the carbon surface (Figure 5c), should not have been oxidized during the synthesis in a highly reducing atmosphere, we suggest that heating up the sample with the Raman laser could have resulted in the (surface) oxidation of tin nanoparticles.

While some tin particles have a spherical or ellipsoidal appearance, others resemble thin plates. Both types may occur simultaneously on the surface of a single aggregate or flake. However, also several examples of soot aggregates and flakes covered by just one of the two types were found. These nanoparticles might form due to the condensation or deposition of tin vapor when the pyrolysis gas and carbon pass the upper reactor section on top of the LM and cool down. Defects in the graphitic carbon lattice have been reported several times as nucleation sites for metal nanoparticles deposited from metal vapor.[^54^, ^55^, ^56^] A connection between carbon defect density and metal nanoparticle formation seems likely, as many pyrolytic carbon flakes and soot aggregates covered in small particles were observed and both types of carbon are characterized by a rather disordered structure, rich in defects. However, no unambiguous example of a CNO covered with tin particles was found. The shape of the particles could depend on the properties of the carbon surface, its defect density and the forces interacting between the carbon surface and the tin. Besides, the local temperature and tin vapor pressure in the upper reactor section might influence whether spherical or plate‐shaped tin particles are formed. The mechanisms resulting in one particle shape or the other require further investigation, which is beyond the scope of this article. The tin nanoparticles appear to be attached to the carbon particles. Therefore, it is unlikely that they can be separated from the carbon in a gas stream or via floatation. Instead, the treatment of the sample with acid, e. g., HCl, and the dissolution of the tin salt seems to be a promising hydrometallurgical approach to remove the tin nanoparticles.

### 3.2 Carbon Formation Mechanisms

**Figure 7:**
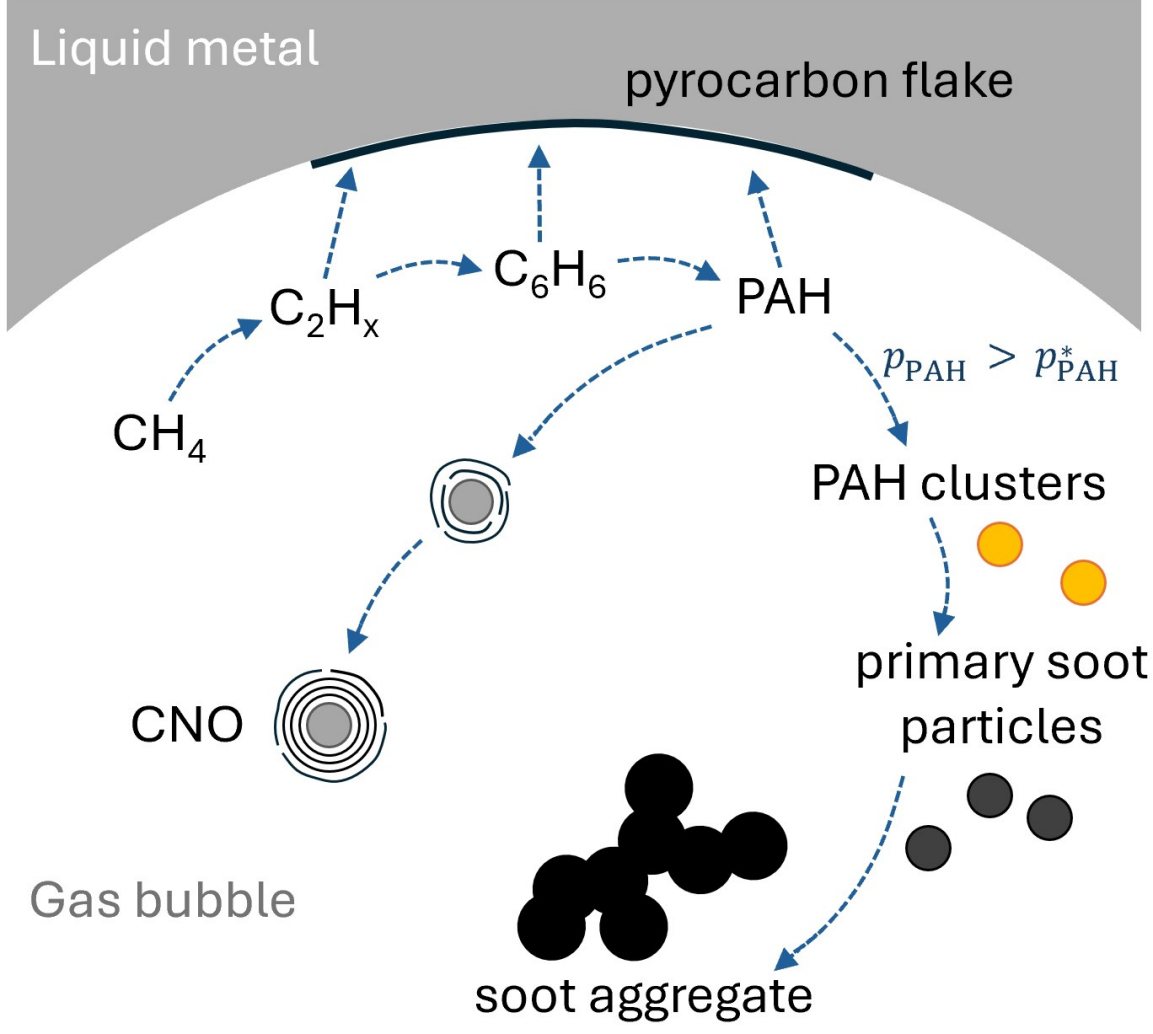
Carbon formation routes inside a gas bubble during methane pyrolysis. Deposition of small hydrocarbons and PAH on the bubble interface results in pyrocarbon flakes, deposition of PAH on particles in CNO. Soot aggregates result from a condensation/ nucleation mechanism involving PAH clusters.

This section discusses the formation mechanisms for pyrocarbon flakes, soot aggregates and carbon onions based on literature and our own hypotheses. Figure 7 gives a schematic overview of the various carbon formation mechanisms we assume to take place inside a gas bubble rising in the hot LM. As Qiao et al.^[57]^ demonstrated that carbon dissolution and diffusion in liquid tin is not observed during methane pyrolysis with tin as molten medium, it is reasonable to assume that carbon formation is limited to the bubbles.

#### 3.2.1 Soot Aggregates

Soot formation proceeds via a complex network of consecutive and parallel reactions, which is shown in a very simplified manner in Figure 7. Details are given in several extensive reviews,[^58^, ^59^] which can be summarized as follows. Starting from methane, the soot precursor molecules increase in size with C_2_H_2_ and benzene as important intermediates. While the hydrocarbons grow, more and more hydrogen is released, thus reducing the atomic H/C ratio of the molecules. This is followed by the formation of small PAH, which keep growing into larger PAH until PAH clusters form. Finally, soot inception results in the formation of small primary particles with diameters of only a few nanometers. While surface growth leads to increasingly large primary particles, collisions of those particles result in soot aggregation.

In the pyrolysis reactor filled with LM, these processes leading to the formation of soot aggregates take place in the bulk gas phase of the bubbles. Without the addition of argon above the LM surface to quench the pyrolysis reactions and to reduce the residence time in the reactor head space, soot formation and/or particle growth might also continue in the gas‐filled upper part of the reactor.

Variations of residence time during the growth stage of soot particles, which result from non‐uniform bubble trajectories as well as coalescence and break‐up of bubbles,^[60]^ might explain the relatively broad primary particle size distribution (PSD, Table 5) determined for the soot aggregates. A further reason for the variation of the particle size could be local differences of the availability of precursor molecules inside a bubble. In contrast to soot particles in the center of a bubble, particles located close to the gas‐LM interface have to compete with the growing carbon flakes on the interface (see Subsection 3.2.2). This could significantly reduce the availability of molecules contributing to soot particle growth close to the bubble interface.

#### 3.2.2 Pyrocarbon Flakes

Inside the LM bubble column reactor employed in this study, the flakes are probably deposited onto the LM surface at the gas‐liquid interface of the gas bubbles, as we elaborated previously.^[34]^ Although we do not know of a study explicitly addressing pyrocarbon deposition on an LM substrate, the growth of graphene on various LM substrates via CVD has been investigated,^[33]^ which shows that LM surfaces can serve as CVD substrates for carbon deposition. Since the LM is an opaque medium, just as solid metal, the bubbles are optically inaccessible and experimental evidence of the carbon formation process is currently not available. An extensive analysis[^61^, ^62^] of the pyrocarbon deposition mechanism from methane and other hydrocarbons showed that already small precursor species such as C_2_H_*x*_ (with *x=*2,4,6) and even methyl radicals contribute to the growth of a solid carbon film. Taking this into consideration, the deposition of carbon on the gas‐LM interface is likely to start almost immediately after a bubble is formed or even during its formation.

De Pauw et al.^[63]^ studied the structural evolution of pyrolytic carbon films deposited via CVD from methane. They described the formation of carbon islands, of which some had an onion‐like structure, on thin layers of pyrolytic carbon. According to their hypothesis, islands with a layered onion structure might form when the nucleation of large molecules starts in the gas phase, as described for soot formation.[^58^, ^59^] De Pauw et al.^[63]^ assume that the collision of such nuclei with the thin pyrocarbon layer already present results in onion‐like carbon islands. De Pauw et al.’s hypothesis could also explain the formation of large islands on some pyrocarbon flakes, which are described in Section 3.1.1. The circular island structures shown in Figure 3d are larger than the islands reported by De Pauw et al.,^[63]^ though. They observed an increase of the average island size for longer residence times. According to our model,^[34]^ the residence time of the bubbles in the LM (approx. 5 s) is about twice as long as the longest residence time tested by De Pauw et al.,^[63]^ which could explain larger islands. As a second competing mechanism De Pauw et al.^[63]^ suggest the growth of islands from active sites on the surface of the pyrocarbon film, which could explain the presence of carbon islands that seem to grow out of the pyrocarbon layer without apparent onion‐like ordering.^[63]^ The latter could be the origin of the smaller dark features scattered across the carbon flake in Figure 3e. They do not exhibit the Moiré effect and are therefore probably less ordered.

Assuming island formation explains the presence of the roughly circular/ hemispherical structures, this still leaves the questions why some carbon flakes are scattered with these structures whereas others exhibit a smooth surface. Several phenomena could be relevant in this context: Gas circulation inside the bubbles^[64]^ might result in preferential impact zones where particles formed by nucleation from the gas phase collide with the thin pyrolytic carbon layer covering the gas‐LM interface. Another factor is the residence time distribution of the rising bubbles, as both coalescence and break‐up of bubbles might occur.^[60]^ Therefore, some bubbles could be too fast for nucleation to start whereas others remain inside the LM long enough for soot nucleation and particle growth to take place

#### 3.2.3 Carbon Onions

The mechanism leading to the formation of CNO in the tin filled bubble column reactor is currently not clear and can only be hypothesized. For the synthesis of CNO from methane usually metal catalysts are employed that dissolve carbon, e. g., Ni or Co,[^45^, ^46^, ^65^] which may result in CNO with metal cores. Due to the decrease of the fusion temperature for nanoparticles, He et al.^[45]^ assumed that at least some of the metal catalyst particles were molten during the growth of CNO. However, many CNO synthesized via this method appeared to have hollow cores, which they attributed to the evaporation of liquid Ni from the core of the CNO after the growth of carbon layers around the metal droplets.^[45]^ In agreement with this theory, Banhart et al.^[66]^ showed that CNO shells were permeable to various types of metal atoms, which they attributed to defects, such as carbon rings with more than six carbon atoms in the spherical graphene layers, screw dislocations and discontinuous shells.

As described previously in Section 3.1, some of the CNO in our samples contain a tin core, for example the one displayed in Figure S6b in the SI. Tin differs from the catalytic metals mentioned before, though, as the carbon solubility in liquid tin is very low and tin is not known to form carbides.^[67]^ Therefore, if tin plays a role in the growth of CNO, the mechanism would probably differ from the previously suggested growth mode that includes carbon dissolution. As suggested by He et al.,^[45]^ the scarcely found CNO with hollow cores might have formed due to the evaporation of a liquid tin core.

Regarding the growth of CNO, several authors[^68^, ^69^] suggest growth routes that involve a vapor‐solid mechanism, where a continuous deposition of carbon species on the surface of CNO and their subsequent ordering results in the formation of new layers. This agrees with our observation that the outermost layers of some CNO, e. g., the one depicted in Figure 5b, appear to be discontinuous. This could mean that the ordered surface promotes the formation of further ordered layers, as in epitaxial growth. For small PAH on a graphite surface, Florio et al.^[70]^ observed an ordered self‐assembly at room temperature under vacuum. Although these conditions are far from the pyrolysis conditions of this study, their results indicate that an ordered graphitic surface might indeed promote an ordered arrangement of adsorbed PAH.

Assuming the growth of new onion layers proceeds in the described manner, the question remains how the first ordered spherical layer is formed, which might then promote the growth of further layers. Based on the previously described observations regarding the various core types of the CNO, we suggest that in a first step, large PAH, which can be regarded as small graphene fragments, and/or fullerene fragments might encapsulate other particles. This is in analogy to the mechanism put forward by Huang et al.^[69]^ to describe CNO formation as a result of benzene pyrolysis. As methane pyrolysis proceeds via many intermediates, including benzene and PAH,[^61^, ^71^] the gas mixture in our experiments could potentially react in a similar way. The core particles could have various natures: PAH clusters or soot primary particles, both expected to form as part of the reaction mechanism leading to soot aggregate formation,[^58^, ^59^] might result in disordered cores of varying diameters. Tin droplets might form during the coalescence or break‐up of rising bubbles and get encapsulated to create CNO with tin cores. Fullerenes or fullerene fragments might also act as an initial ordered substrate surface.

Crowley et al.^[72]^ reported the pyrolytic synthesis of fullerenes from various PAH including naphthalene, which is an intermediate product of CH_4_ pyrolysis.[^59^, ^61^] Besides, we presently cannot rule out that tin vapor is involved in the initial formation step of CNO. Huang et al.^[69]^ describe an arc‐discharge process, in which the simultaneous formation of carbon and metal (Ni) vapor is an important step for the formation of CNO. Due to the many possibilities, the suggested mechanism for the formation of large CNO in a liquid metal filled methane pyrolysis reactor requires further investigation as part of future studies.

Some authors[^73^, ^74^] also mention a favorable effect of nitrogen on CNO formation: Nitrogen atoms tend to be integrated into the hexagonal graphitic lattice by the formation of pentagons, which are crucial for the growth of spherical carbon layers.^[49]^ He et al.^[73]^ and Czigany et al.^[74]^ observed that the incorporation of N promoted the growth of spherical carbon layers. The degree of curvature in CNO layers correlated to the amount of incorporated N, i. e., higher N content for inner layers.^[74]^ During the synthesis of all carbon samples of this study, nitrogen was present in the reactant gas mixture. XPS analysis of those samples did not indicate nitrogen incorporation for the surface layers. However, a preferential incorporation of N in the inner layers of the large CNO (several 100 nm in diameter), might not have been detectable by XPS (information depth about 10 nm). CHN analysis was not clear regarding the incorporation of N. For samples C#1 to C#4, which all contain CNO, traces of N were detected that only slightly exceeded the blank analysis (see Table S2 in the SI). According to those results, the ratio of N/(C+N) would range from 1 to 4 at %, which would agree with ranges reported in other studies.[^73^, ^75^] However, the sensitivity of CHN analysis was impeded by the high mass fractions of tin and N contents similar to blank values should not be taken as unambiguous evidence for the presence of nitrogen in the samples. A repeated CHN analysis of C#2 six weeks later did not detect N, making either N loss upon aging of the sample or an erroneous detection of N during the first set of analyses likely. A repetition of the synthesis experiments with argon instead of nitrogen as part of a future study could help to elucidate the potential role of nitrogen during methane pyrolysis in an LM filled bubble column reactor.

### 3.3 Influence of Process Conditions

This section analyzes the influence of the synthesis conditions on the properties of the solid carbon product. The effects on the overall pyrolysis performance, regarding conversions and yields, are the subject of Part 1 of this study and the respective analysis can be found elsewhere.^[27]^ The focus of this section lies on how the pyrolysis temperature and the initial methane volume fraction of the feed gas affect which types of carbon are formed. The analysis encompasses the carbon flakes, soot aggregates and CNOs, which appear to be the three most prevalent carbon morphologies.

**Figure 8:**
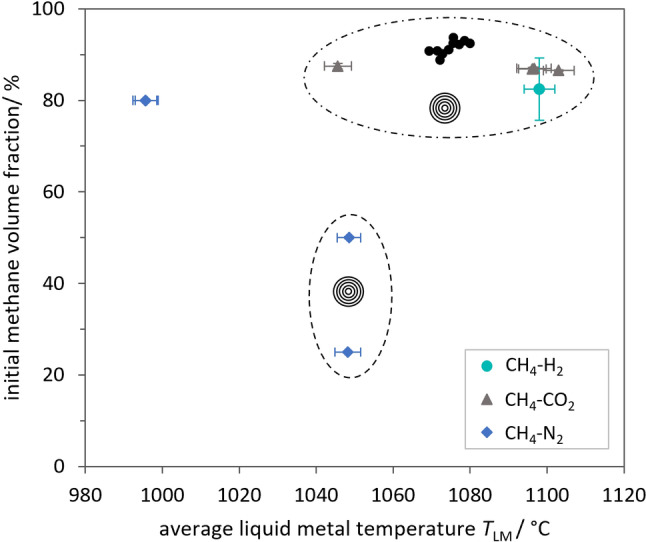
Types of carbon found in samples synthesized at various liquid metal temperatures and from feed gas with various volume fractions of methane. The residence time of the gas bubbles in the liquid metal is estimated at approximately 5 s based on model calculations.^[34]^ Concentrical circles indicate the presence of carbon onions, and the image of a soot aggregate indicates the presence of soot aggregates in the respective samples. All samples contain carbon flakes.

Figure 8 visualizes which types of carbon were identified in the samples for the respective combinations of average LM temperature and initial methane molar fraction. Carbon flakes were present in all samples. Therefore, they are not marked by a specific symbol or area. Synthesis conditions resulting in the formation of CNO and soot aggregates are indicated by stylized depictions of the respective carbon species. Whether further types of carbon were found in the samples was previously listed in Table 3.

In the coupled CCU process, the methanation unit can be run either with H_2_ or with CO_2_ surplus as elaborated in Part 1.^[27]^ The resulting product gas mixture, which is fed into the pyrolysis unit, then contains either H_2_ or CO_2_ as most important reactive side component. However, the following analysis neglects whether only nitrogen or also other gases such as H_2_ or CO_2_ were present in the feed gas mixture. As can be seen from Figure 8, the main carbon species were the same when either H_2_ or CO_2_ were present in the reactant gas. XPS analysis of two samples (Figure S9 and Table S3 in the SI) also confirmed that there is no noticeable difference between the surface composition of carbon synthesized in the presence of either CO_2_ or H_2_. Temperature and methane concentration are therefore seen as the main influencing factors affecting the carbon product.

Several samples were synthesized from about 80 to 90 vol.% methane at an LM temperature of approximately 1000 °C, 1050 °C and 1100 °C. While at the lowest temperature only flakes are found in the samples, at both higher temperatures also CNO and soot aggregates are synthesized. Although only carbon flakes were observed for the samples obtained at 1000 °C, a few flakes seemed to be covered in small dark dots, only a few nanometers in diameter (Figure S5a in the SI). These might be the equivalent to the larger carbon islands found on carbon flakes of other samples. Due to the lower reaction temperature, soot particle growth or aggregation might not have taken place, but the pyrolysis reactions could have progressed to the stage of PAH cluster formation or even soot nucleation. Nascent soot particles with diameters in a similar range to the dots observed on the surface of some flakes have been reported.^[58]^ The collision of carbon flakes with these small particles, or the PAH clusters preceding their formation, could be the origin of the dotted flakes. The synthesis of only flakes at 1000 °C is a huge achievement regarding product quality as it eliminates the necessity to separate various types of nanocarbon in an additional process step. However, this comes at the cost of very low carbon yields (<1 %^[27]^). A techno‐economic analysis, which is beyond the scope of the present study, would have to show if the advantage of producing a single type of carbon outweighs this drawback.

Another look at Table 5, which shows the PSD of the primary particles of the soot aggregates, gives rise to the assumption that at 1050 °C CNO might prevail, while soot formation seems to increase when the temperature rises to 1100 °C. This is indicated by the low number of only *n=*15 primary soot particles that were analyzed for sample C#4 (1050 °C). This was due to the rare occurrence of soot during TEM analysis of this sample, which was contrasted by frequent sightings of CNO in the sample (ratio 5.87 of primary particles CNO:soot). CNO were also present in the other samples synthesized at 1100 °C as listed in Table 3 and shown in Figure 8. However, in those other samples, soot particles appeared to be more abundant than in sample C#4 (ratio CNO:soot ranging from 0.06 to 0.22). An increased soot yield at a higher reaction temperature would be in agreement with other studies on soot formation.[^76^, ^77^] The particle number ratios determined by image analysis should not be seen as an exact quantification, though, for which a more extensive analysis would be required. However, the large difference in the ratios determined for the two temperature levels indicates a tendency for increased soot formation, relative to CNO formation, at higher temperatures.

Regarding the initial methane volume fraction, a comparison of the samples synthesized at 1050 °C shows that only for the highest concentration of 87.49±0.62 vol.% soot aggregates were found. Both 25.00±0.14 and 50.00±0.19 vol.% of methane only resulted in the growth of carbon flakes and CNO at 1050 °C. In analogy to rising methane concentrations, a transition from flakes and CNO to the additional formation of soot aggregates might also exist for an increase in temperature, which we hypothesize to take place between 1000 °C and 1050 °C for about 80 vol.% methane. Similarly, further lowering the initial methane concentration might lead to the growth of only flakes, in analogy to a decreasing LM temperature. These hypotheses are based on the assumption that for the calculated residence time of approximately 5 s, slow reaction rates as a result of either relatively low temperature or low methane concentration only result in flake growth via CVD on the bubble interface. In agreement with observations from literature, which were discussed in Section 3.2, only small precursor molecules would be needed for CVD growth of pyrocarbon flakes. Increasing the reaction rate by either increasing the temperature or methane concentration allows the pyrolysis reactions to advance further to the stage of PAH formation and growth. The literature and theories presented in Section 3.2 regarding possible routes for CNO formation give rise to the assumption that larger PAH or fullerene fragments are required for the formation and growth of CNO.

Regarding soot formation, the partial pressure of certain PAH might play an important role. Martin et al.^[58]^ assume that soot inception takes place via a combination of chemical and physical processes. For a homogeneous nucleation process supersaturation of the gas phase with certain PAH would have to be achieved. Diluting the reactant gas with an inert gas, e. g. nitrogen, would reduce the overall partial pressure of reactive species. This could explain why no soot aggregates were found in samples C#5 and C#6, which were synthesized at 1050 °C from a gas mixture containing only 25 vol.% and 50 vol.% CH_4_ respectively. Due to the dilution soot inception might be prevented or at least delayed. Slow reaction rates at low pyrolysis temperatures would have a similar effect as it would take longer for large molecules to form in sufficiently large amounts. At the same time, the competing carbon formation mechanisms for the growth of flakes and CNO consume soot precursor molecules, thus counteracting the build‐up of PAH partial pressure. Therefore, the formation of large soot precursor molecules has to take place fast enough to compensate for their consumption in surface reactions and to reach a critical partial pressure within the given residence time. This can be achieved by either increasing the initial methane concentration and/or the pyrolysis temperature. In agreement with this, Figure 8 shows that soot aggregates were only found in samples synthesized at high temperatures from reactant gas mixtures with high methane concentrations.

All in all, the findings and hypotheses presented in this section and visualized in Figure 8 are plausible in the context of the available literature, which was discussed in the previous Section 3.2 for the three prevailing types of carbon. Nonetheless, further experiments with variations of temperature and methane concentration are required to expand the available dataset and to test the hypotheses for more synthesis conditions.

### 3.4 Carbon Utilization

This section explores potential applications for the carbon products synthesized via the CCU process with the laboratory‐scale pyrolysis reactor described above. For the utilization of individual carbon species, the removal of the tin particles and the isolation of those species would be necessary. In case of tin removal and carbon separation or after a process scale‐up, the carbon characterization should be repeated to analyze possible effects on the carbon product. An analysis of recent research activities addressing the use of solid carbon products has revealed several promising fields. Rough estimates of the amounts of carbon that could potentially be used in the applications are given. The respective prices are only rated as high or low based on the field of application, as to the best of our knowledge there are no readily available equivalents for pyrocarbon flakes or large CNO on the market. Besides, several of the applications are still not fully developed and the price range for carbon products spans several orders of magnitude.^[21]^ Any more precise price estimates would therefore be highly speculative. It should also be noted that without actual tests of the carbon in applications to determine the performance, both the estimated amounts of carbon and the prices are purely hypothetical. The underlying assumption is that the carbon would be suitable for the respective application. The price customers might be willing to pay would strongly depend on the performance compared to reference materials that are already on the market, the reduction in CO_2_ emissions compared to those reference products and the CO_2_ price.

Large CNO might be used as lubricants,^[50]^ while smaller CNO have also shown promising results as a material for supercapacitor electrodes.[^78^, ^79^] The graphene‐like pyrocarbon flakes might be suitable to tune the gas permeability of membranes towards higher or lower permeability, as reported for graphene composite materials.[^80^, ^81^, ^82^] The observed effects were attributed to the high aspect ratio, large surface area and low gas permeability and to a changed arrangement of the polymer chains, respectively.^[82]^ Regarding the inherent material properties, pyrocarbon is used as a gas diffusion barrier, e. g., in nuclear applications,^[83]^ due to its low gas permeability. The utilization of the thin pyrocarbon flakes to tune the gas permeability of membranes might therefore achieve similar results as those reported for graphene composites. The applications suggested in this paragraph are seen as specialty applications that use relatively small amounts of carbon (absolute) but might pay high prices.

Concerning the mixture of carbon materials characterized in this study, mostly promising tests with other carbon nanomaterials[^84^, ^85^, ^86^, ^87^, ^88^] imply that the application of the carbon powder for soil enhancement might be an option and is worth investigating. In future studies, special attention should be paid to potential adverse effects^[89]^ on plants or soil microorganisms, though, to gain a holistic picture of the advantages and risks of adding the carbon powder to soil. The observed effects (positive and/or negative) might also differ strongly, depending on the types of soil, types of carbon, application rate and plants tested.[^90^, ^91^] In the context of biochar soil amendment, Tenic et al.^[91]^ drew the conclusion, that a prediction of the outcome of biochar addition was almost impossible because of the complex interactions of the system components. A risk mentioned several times in the context of biochar, which is produced via biomass pyrolysis, is the potential contamination of pyrolytic carbon with PAH.[^84^, ^86^] The same holds true for solid carbon products obtained from any methane pyrolysis process (not limited to molten media), as PAH are known intermediates of methane pyrolysis.[^58^, ^59^, ^61^, ^71^] An analysis of the PAH content – and, if necessary, a thorough cleaning procedure – are therefore crucial before applying any pyrolytic carbon to soil. Lorentz and Lal^[92]^ also point out the risk that small particulate matter (PM10 and PM2.5) could get airborne during the application process. This would cause pollution and could even have a negative effect on global warming.^[92]^ Therefore, this aspect should be kept in mind when the application of carbon nanoparticles is considered. Lorentz and Lal^[92]^ mention yet another potentially adverse effect on the climate. The addition of dark particles to soil can reduce its albedo. As a result, less solar radiation is reflected into space.^[92]^ At first, soil enhancement seems like an application with the potential to use and store huge amounts of carbon from the CCU process we describe or from the pyrolysis of natural gas. Often, carbon application rates of several tons per hectare are suggested.^[91]^ With these rates and approximately 1.5 billion hectares of agricultural crop land,^[93]^ billions of tons of carbon could theoretically be used for soil enhancement. The literature on the utilization of nanocarbons is limited, though, with most studies focusing on biochar. The complexity of finding suitable systems and understanding the interactions of the system components would make extensive studies with any new type of carbon necessary. Potentially adverse effects on microorganisms, the vegetation or the climate might limit the areas where carbon from methane pyrolysis might be used beneficially for soil amendment. An estimation of the volume of carbon from methane pyrolysis that could be used for this application is therefore difficult. Regarding the carbon price, several studies[^90^, ^92^] remark that it would have to be low enough that the increased crop yields resulting from the soil amendment would make the carbon application profitable for farmers.

Applications requiring a combination of carbon and tin are of special interest, as this might simplify the product cleaning process significantly. Carbon with a high defect density could be a suitable anode material for sodium ion batteries (SIB), while the tin nanoparticles scattered on its surface could increase the capacities of batteries (both Li and Na) via alloy formation.^[94]^ Strategies to overcome the challenge of volume expansion upon sodiation^[95]^ and lithiation^[96]^ include the reduction of Sn particle size and their dispersion in a carbon matrix to provide volumetric buffer space and to prevent aggregation.^[94]^ Typically, hard carbon (HC) is studied as a carbonaceous anode material for SIB.[^97^, ^98^] HRTEM analysis of HC reveals both curved and straight clusters of few aligned graphene layers,^[99]^ similar to structures found in our pyrocarbon flakes. The markets for SIB and lithium ion batteries are expected to grow strongly within the next years as increasing the storage capacity for electrical energy is a crucial part of the energy transition. Accurate predictions about the future price of HC or the volume of HC that will be needed for SIB production are difficult, though, as the SIB market is still in an early stage of development.^[21]^ If the combination of carbon and tin nanoparticles that we obtain as product of the CCU process proves advantageous in SIB applications, this could potentially boost the price.

Besides, the combination of carbon and tin or tin oxide nanoparticles could be of interest for catalytic applications, e. g., in the context of residual biomass utilization.[^100^, ^101^, ^102^] Furthermore, Zhao et al.^[103]^ observed a high catalytic activity of SnO_2_ in methanol steam reforming, although no carbon supports were tested. The utilization of the carbon as a catalyst falls in the area of specialty applications where potentially high prices could be achieved. However, it would probably use only relatively small amounts of carbon compared to other applications.

Cement and concrete production could be another promising application to achieve long‐term carbon storage on top of large‐scale carbon utilization, thus fully exploiting the CCUS potential of the process. Research on concrete enhancement by the addition of graphene‐like carbon materials (graphene oxide, graphene, graphene nanoplatelets) has received a lot of attention recently, as summarized in various reviews.[^104^, ^105^, ^106^, ^107^] The mechanical strength, durability and further properties of cementitious materials were enhanced significantly by the addition of nanomaterials (not limited to carbon.[^100^, ^108^] In many of those tests, the mass proportion of graphene‐like materials was very low (<0.1 %), though.^[106]^ Assuming this mass ratio, with the current global production of approximately 4.1 billion metric tons of cement,^[109]^ about 4.1 million tons of carbon could be used to enhance cement. If small amounts of carbon additive resulted in significantly improved concrete characteristics and reduced the required amount of building material, reasonably high prices could be expected.

## 4 Conclusions

A CCU process that couples direct air capture (DAC), catalytic methanation and methane pyrolysis to produce solid carbon materials from atmospheric CO_2_ was successfully demonstrated. A bubble column reactor filled with liquid tin was used for the pyrolysis step and pneumatic conveying was employed to continuously remove the solid carbon powder from the pyrolysis reactor. Results regarding the performance of the overall process and the individual process steps are the subject of Part 1^[27]^ of this study. The present article, Part 2, presents a thorough analysis of the thus obtained solid carbon product. Four carbon samples synthesized from atmospheric CO_2_ during the coupled process operation under various synthesis conditions and four additional samples obtained from individual pyrolysis experiments are characterized. The analysis with SEM, TEM and Raman spectroscopy revealed soot aggregates, large carbon (nano−)onions (CNO), clusters of small CNO‐like structures, very thin pyrocarbon flakes and elongated closed carbon structures we call “carbon microbes”. The prevailing carbon species seem to be CNO, flakes and soot aggregates. HRTEM and Raman analyses of a sample containing only thin carbon flakes confirm the turbostratic and disordered structure of the pyrocarbon flakes. At the atomic level, the thin flakes therefore differ strongly from other crystalline flake‐like carbon morphologies, i. e. graphite or graphene. The overall very low crystallinity of the carbon flakes and of the other samples containing several carbon species is confirmed by XRD analyses. XPS analyses show the sp^2^‐hybridization of the carbon powders but no differences in the surface composition of a sample obtained from H_2_‐containing methane and another one synthesized from CO_2_^−^ and CO‐containing methane. Tin nanoparticles with spherical and plate shapes were found to cover the surface of some soot aggregates and pyrocarbon flakes.

While H_2_, CO or CO_2_ as side components in the reactant gas do not affect the solid pyrolysis product, the synthesis temperature and the initial methane concentration have a strong influence on carbon formation. They can therefore be used to tune the carbon product composition to obtain either exclusively pyrocarbon flakes, flakes mixed with CNO or a mixture of flakes, CNO and soot aggregates.

For the estimated residence time of approx. 5 s, a slow pyrolysis reaction rate (at 1000 °C) results in the growth of pyrocarbon flakes only via chemical vapor deposition (CVD) on the gas‐LM interface of the bubbles. Increasing pyrolysis temperatures (1050 °C and 1100 °C) lead to additional CNO and soot aggregate formation, with higher temperatures seemingly increasing the ratio of soot to CNO. Reducing the reaction rate at 1050 °C by lowering the initial methane concentration suppresses the growth of soot aggregates, leading to a mixture of pyrocarbon flakes and CNO. For CNO formation, a mechanism including a core particle (e. g., tin, fullerene, nascent soot) and ordered surface growth was identified as most likely. Soot inception is seen as a combination of chemical and physical processes, the latter including nucleation. This would require higher partial pressures of nucleation species than the heterogeneous surface growth of CNO, explaining why a reduction of the initial methane concentration suppressed soot formation.

Based on the carbon properties, sodium ion batteries, super‐capacitors, catalysts and composite materials are seen as potential applications, both for the as‐synthesized mixtures of nanomaterials as well as for individual carbon species.

## Conflict of Interests

The authors declare no conflict of interest.

5

## Supporting information

As a service to our authors and readers, this journal provides supporting information supplied by the authors. Such materials are peer reviewed and may be re‐organized for online delivery, but are not copy‐edited or typeset. Technical support issues arising from supporting information (other than missing files) should be addressed to the authors.

Supporting Information

## Data Availability

The data that support the findings of this study are available in the supplementary material of this article.
