# Supplementary material for: A Carbon Capture and Utilization Process for the Production of Solid Carbon Materials from Atmospheric CO2 – Part 2: Carbon Characterization
Source: ChemSusChem. 2024 Nov 19;18(5):e202401780. doi: 10.1002/cssc.202401780 (PMC11874684; doi:10.1002/cssc.202401780)
Supplement: Supplementary file 1 — Supporting Information [file CSSC-18-e202401780-s001.pdf]

ChemSusChem

Supporting Information

A Carbon Capture and Utilization Process for the Production of Solid Carbon Materials from Atmospheric CO_2 – Part 2: Carbon Characterization

Neele Uhlenbruck,* Benjamin Dietrich, Stefan Heißler, Christoph M. Hofberger, Ralf Krumholz, Leonid Stoppel, Vanessa Trouillet, Peter G. Weidler, and Thomas Wetzel

A Carbon Capture and Utilization Process for the Production of Solid Carbon Materials from Atmospheric CO_2 – Part II: Carbon Characterization

Supplementary Information

Neele Uhlenbruck*[a], Benjamin Dietrich[b], Stefan Heißler[c], Christoph Hofberger[a], Ralf Krumholz[a], Leonid Stoppel[a], Vanessa Trouillet[d], Peter G. Weidler[c], Thomas Wetzel[b]

[a] N. Uhlenbruck, C. M. Hofberger, R. Krumholz, Dr. L. Stoppel
Institute for Thermal Energy Technology and Safety
Karlsruhe Institute of Technology
Hermann-von-Helmholtz-Platz 1, 76344 Eggenstein-Leopoldshafen
E-mail: neele.uhlenbruck@kit.edu

[b] Dr. B. Dietrich, Prof. Dr. T. Wetzel
Institute of Thermal Process Engineering
Karlsruhe Institute of Technology
Kaiserstr. 12, 76131 Karlsruhe

[c] S. Heißler, Dr. P. G. Weidler
Institute of Functional Inferfaces
Karlsruhe Institute of Technology
Hermann-von-Helmholtz-Platz 1, 76344 Eggenstein-Leopoldshafen

[d] V. Trouillet
Institute for Applied Materials (IAM-ESS) and Karlsruhe Nano Micro Facility (KNMFi)
Karlsruhe Institute of Technology
Hermann-von-Helmholtz-Platz 1, 76344 Eggenstein-Leopoldshafen

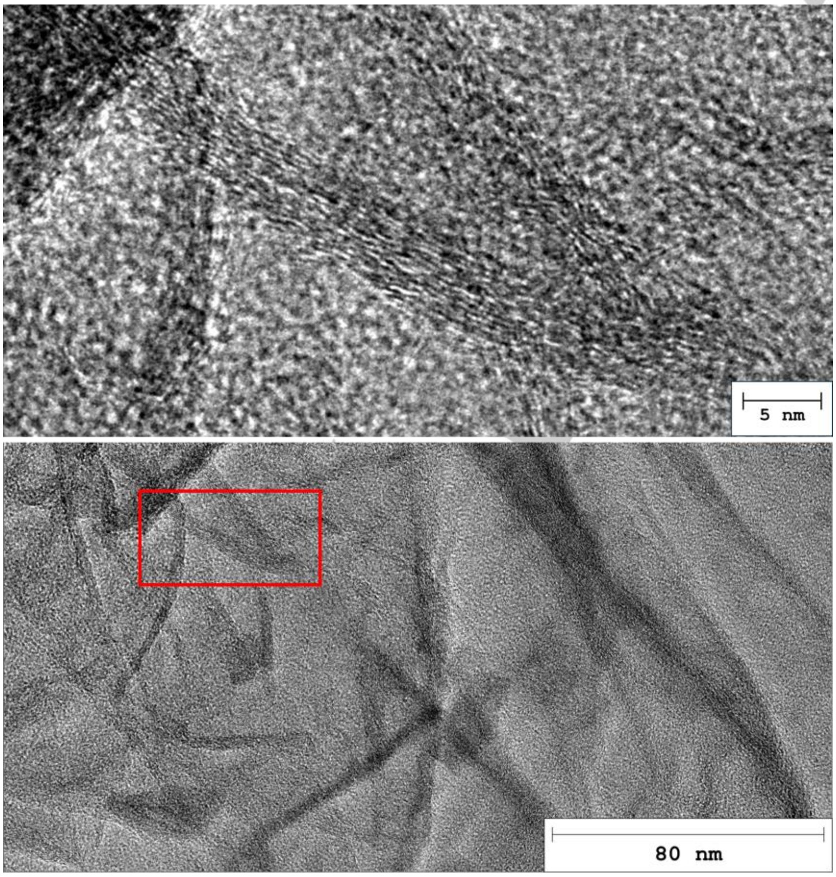

Figure S1. HRTEM image of a carbon flake (bottom) and magnification of the area marked by the red box (top).

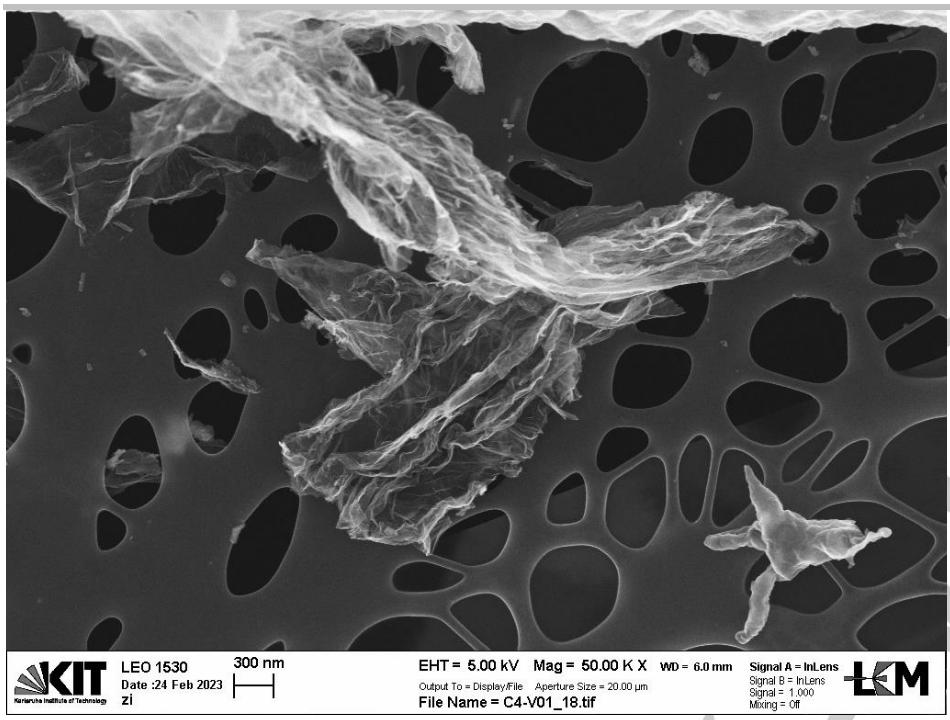

Figure S2. SEM images of a carbon "microbe" (bottom right) and a folded carbon flake (center).

WILEY-VCH

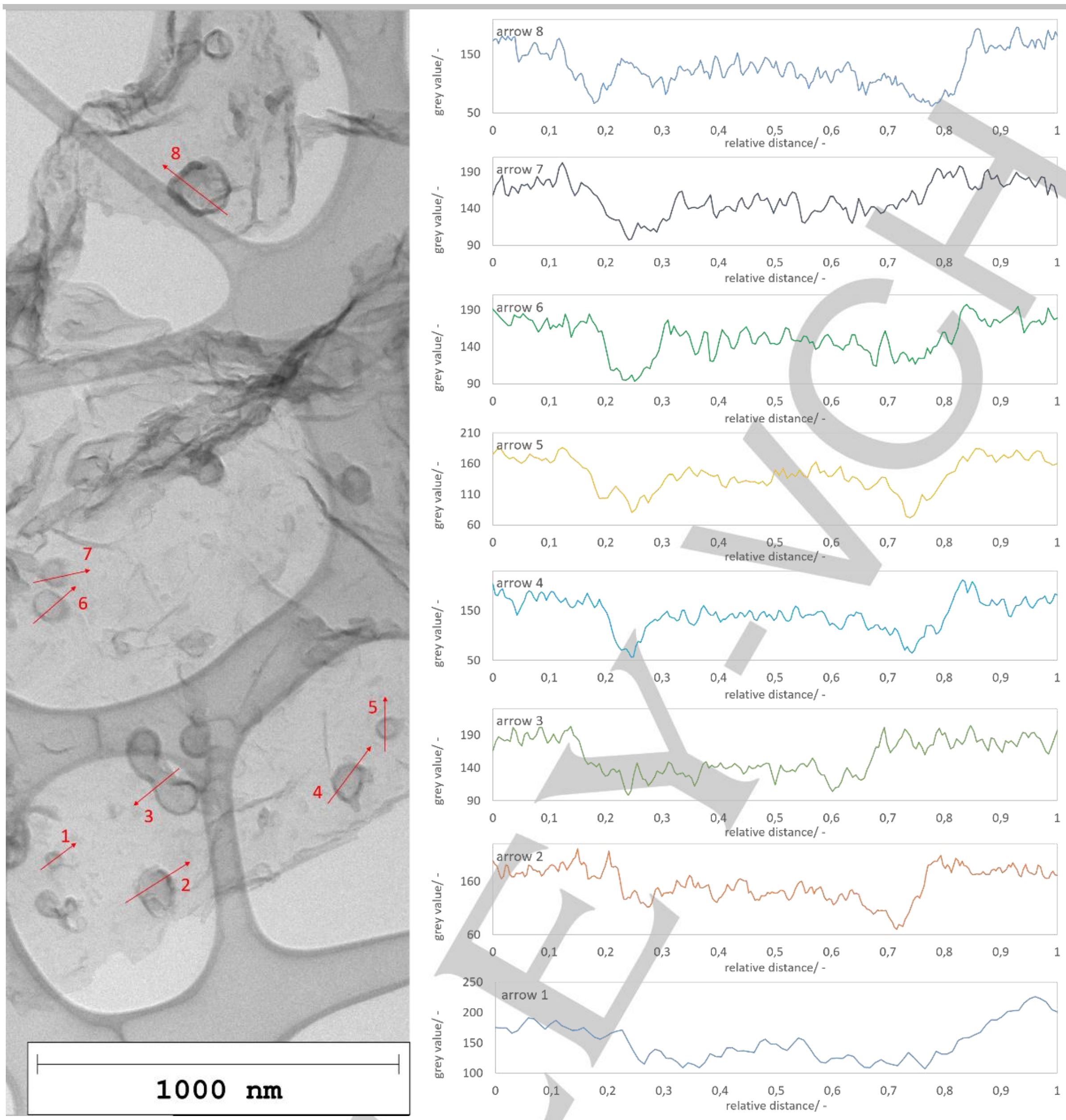

Figure S3. Section of HRTEM image shown in figure 2 b with arrows indicating position and direction of grey value profiles plotted on the right.

WILEY-VCH

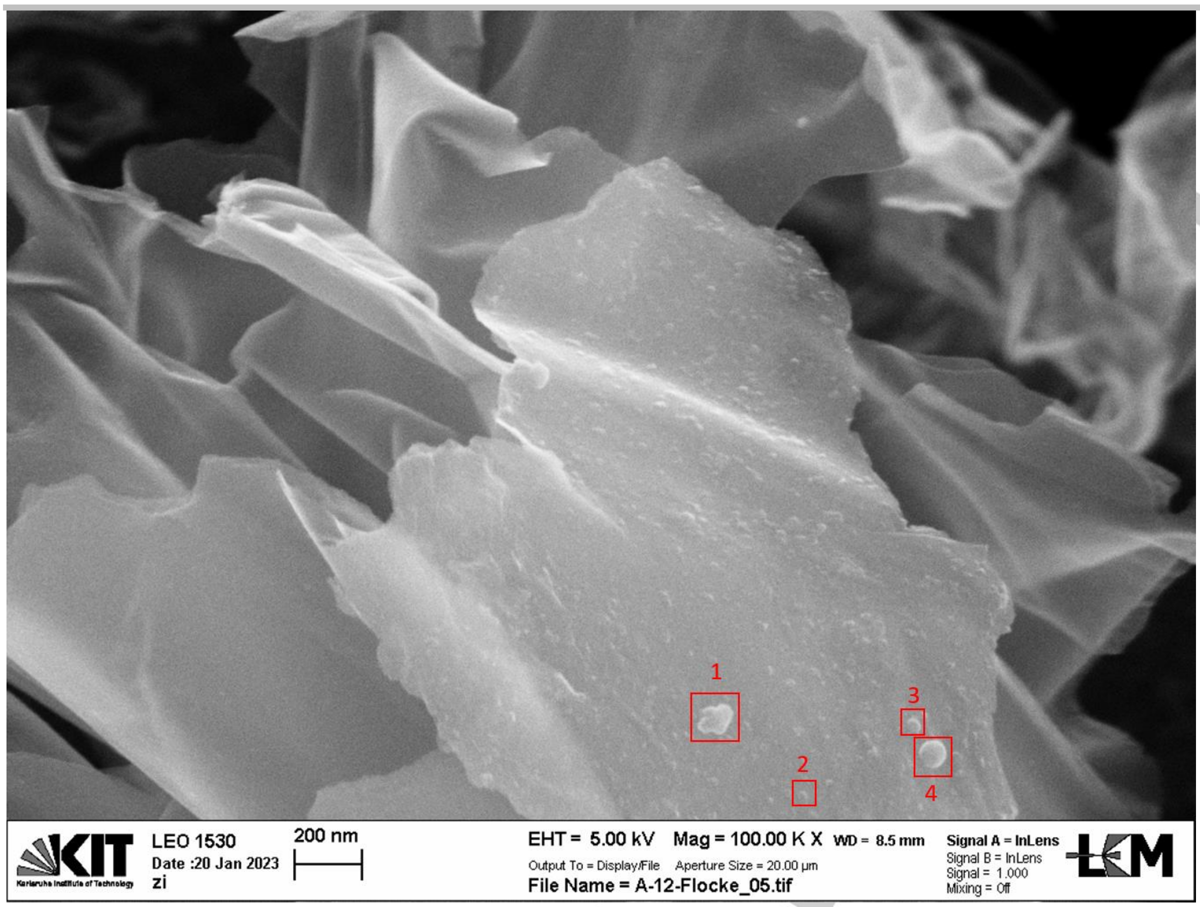

Figure S4. SEM image showing carbon flakes with smooth surfaces in the background and a flake with rough surface in the foreground.

Table S1. Minimum and maximum diameters measured for roughly spherical structures on the surface of the carbon flakes shown in Figure S3 and Figure S4. Numbers # refer to numbers depicted in the images.

#	Figure	Diameter max/ nm	Diameter min/ nm
1	S3	49	49
2	S3	124	108
3	S3	271	89
4	S3	106	88
5	S3	62	61
6	S3	99	91
7	S3	76	74
8	S3	168	156
1	S4	118	79
2	S4	32	29
3	S4	42	37
4	S4	84	79

WILEY

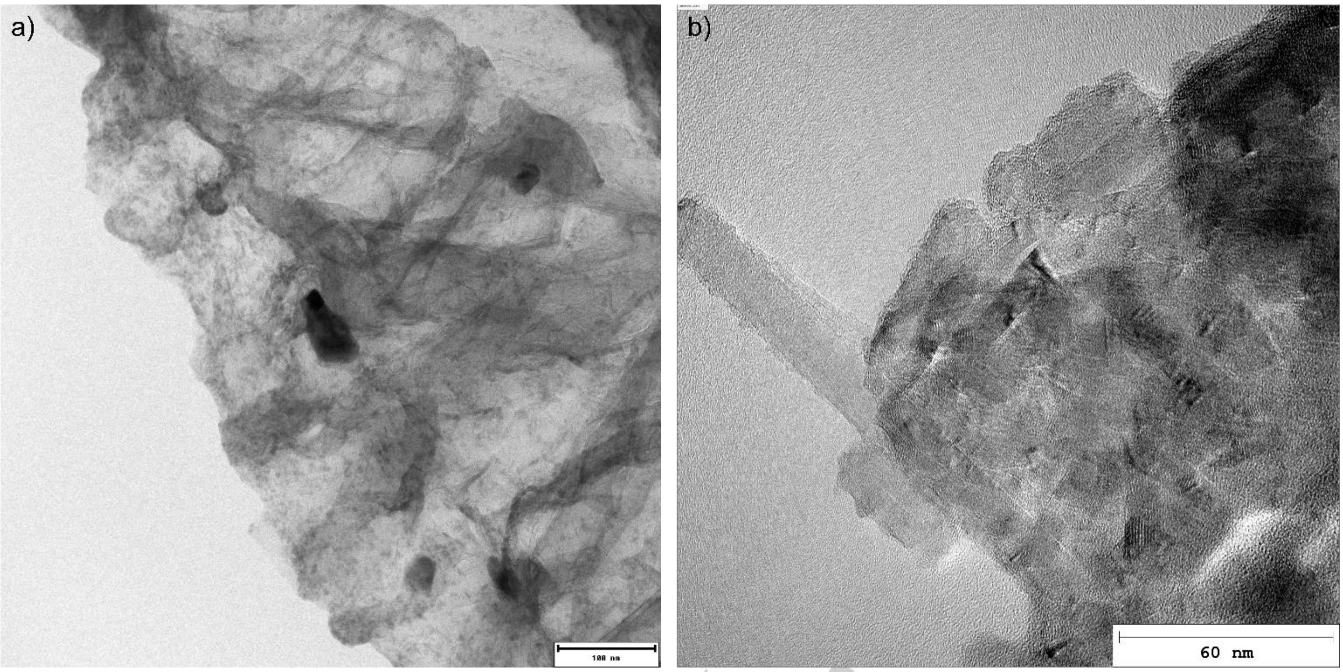

Figure S5. (HR)TEM images of a) a dotted carbon flake and b) a cluster of small CNO/MWCNT-like structures.

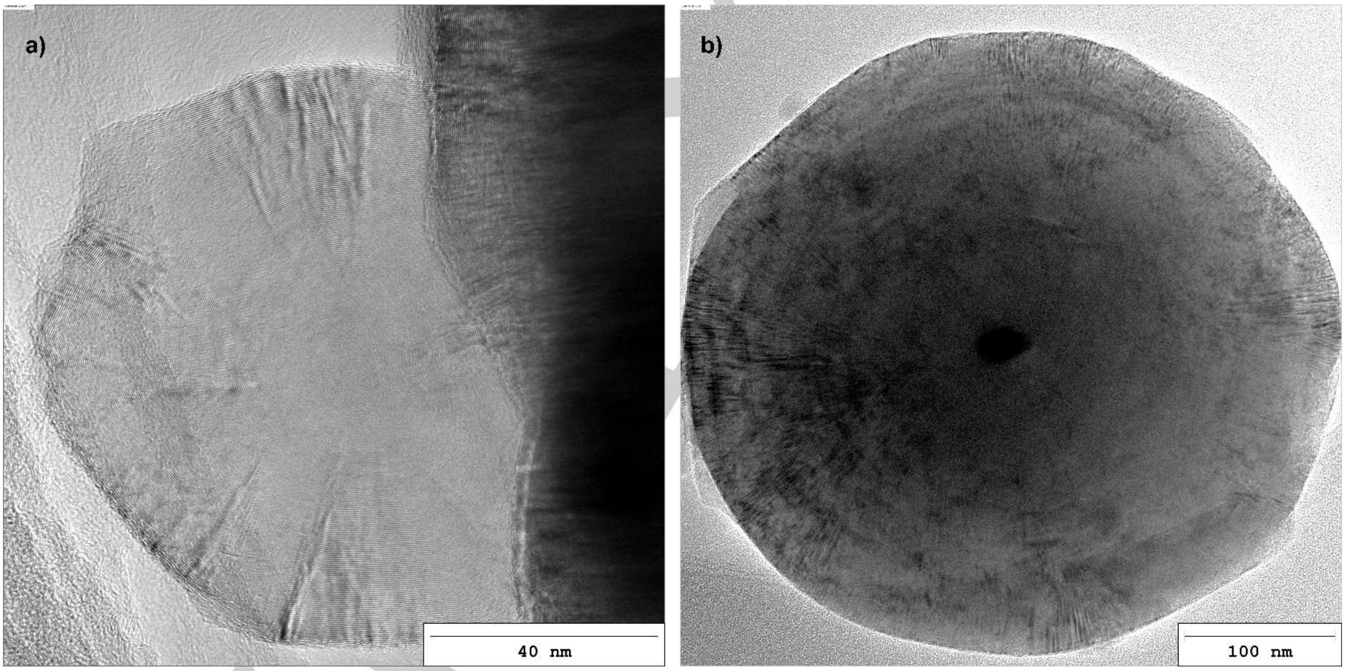

Figure S6. TEM images of carbon onions with a) an amorphous core and b) a tin core.

Table S2. CHN elemental analysis of the samples synthesized from atmospheric CO_2 as given in the SI of Part 1 [25]. The nitrogen blank value was 0.4-0.5 wt.%.

Sample	C in wt.%	H in wt.%	N in wt.%	Sn in wt.% (by difference)
#1 – 1100°C, CH_4-H_2	19.2 ± 2.0	<0.2	0.60 ± 0.06	80.2
#2 – 1100°C, CH_4-CO_2	19.3 ± 0.1	<0.2	0.56 ± 0.07	80.1
#2 – 1100°C, CH_4-CO_2*	19.3 ± 1.1	<0.2	<0.2	80.7
#3 – 1100°C, CH_4-CO_2	15.0 ± 0.6	<0.2	0.54 ± 0.01	84.5
#4 – 1100°C, CH_4-CO_2, 0.5Ar	61.9 ± 0.9	<0.2	0.69 ± 0.04	37.4
#5 – 1050°C, CH_4-CO_2	16.3 ± 0.4	<0.2	0.57 ± 0.05	83.1

*Repetition of analysis six weeks later with a nitrogen blank value of 0.1-0.2 wt.%

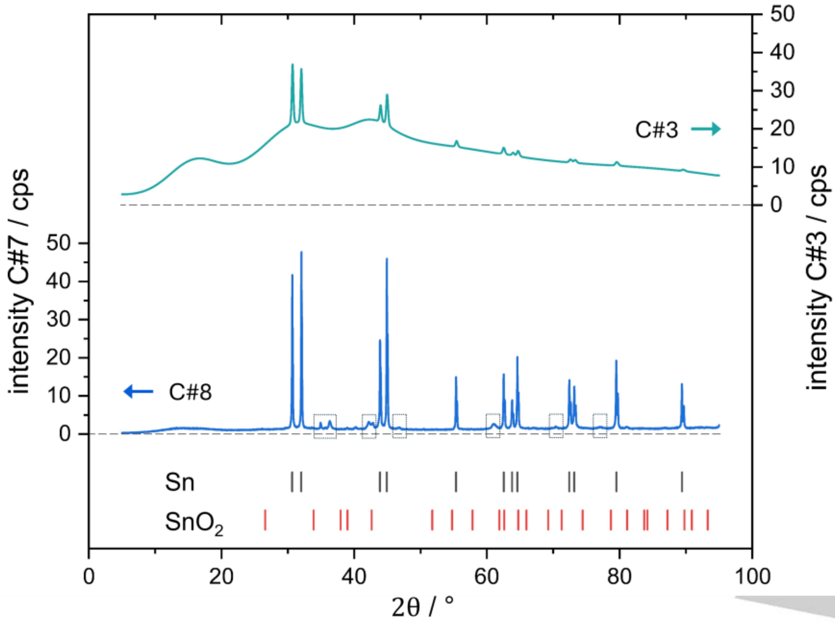

Figure S7. XRD diffractogram of samples C#8 and C#3 without background correction. The small peaks framed by dotted boxes in the diffractogram of C#8 match reference 2θ positions for oxidized iron (+ steel alloy elements) best and originate from the corrosion of a stainless-steel tube.

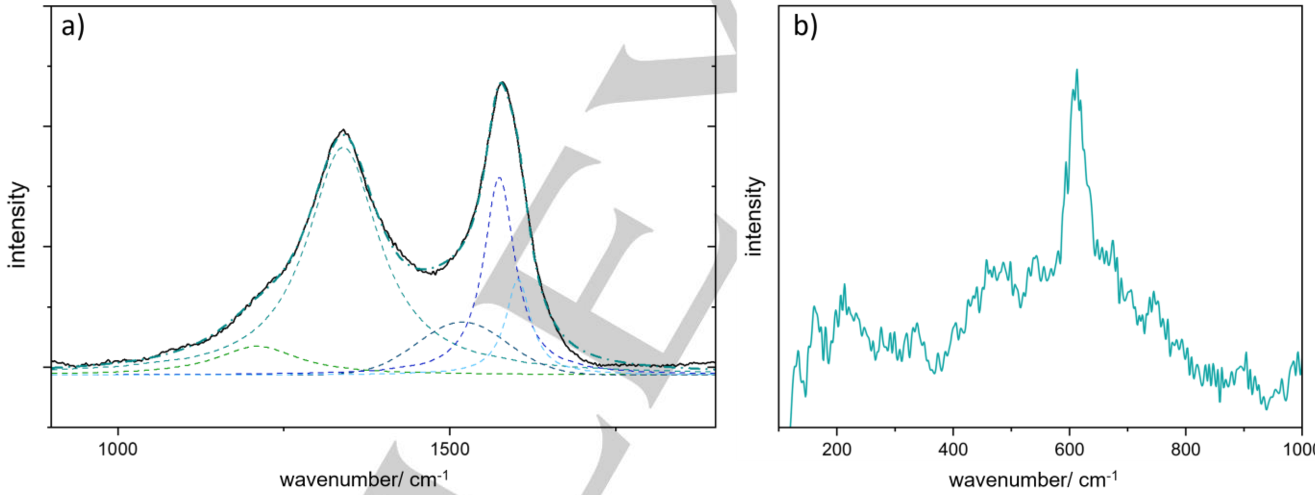

Figure S8. Averaged Raman spectrum of samples C#8 with peak fit according to Sadezky et al. [33] (a) and an example of intermediate frequency phonon modes (IFM) probably originating from SnO_2 nanoparticles as reported in [69] (b).

WILEY-VCH

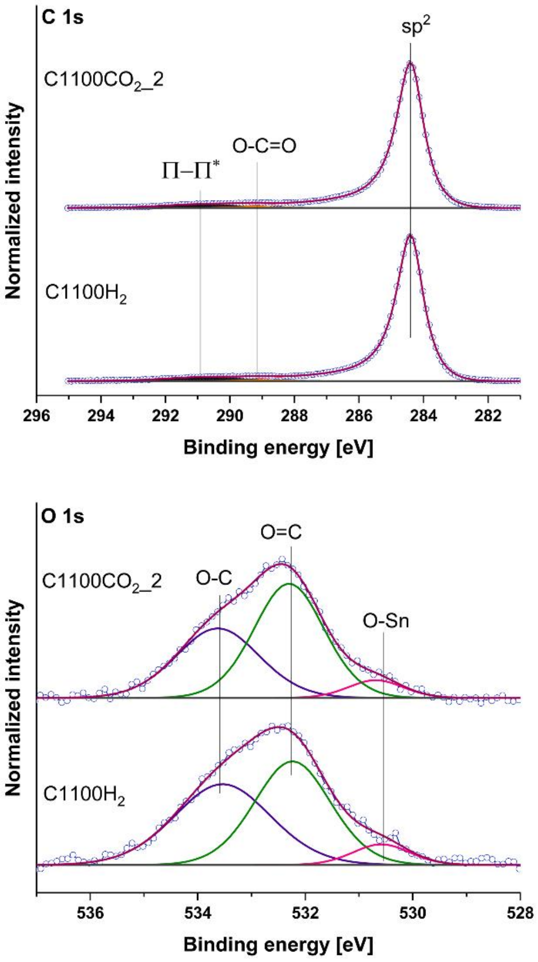

Figure S9. C 1s and O 1s XPS spectra of samples C#1 and C#3.

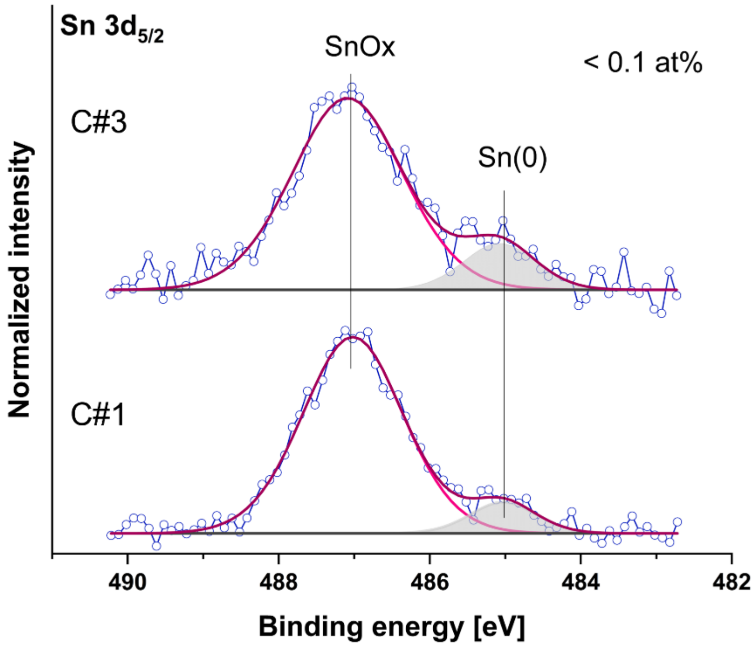

Figure S10. XPS Sn 3d$_{5/2}$ spectra of samples C#1 and C#3

Table S3. XPS Analysis of C#1 and C#3

Photoelectron line	Species	Binding Energy eV	C#1 at.%	C#3 at.%
Si $2p_{3/2}$		102.0	0.2	0.1
C 1s	sp^2	284.4	92.7	93.0
	O-C=O	289.1	0.5	0.5
	π-π*	290.7	4.3	4.0
Sn $3d_{5/2}$	Sn(0)	485.0	0.01	0.01
	SnO_x	487.0	0.07	0.06
O 1s	O-Sn	530.6	0.2	0.15
	O=C	532.2	1.1	1.2
	O-C	533.5	1.0	0.9

WILEY-VCH
